# Antibacterial Activity of Squaric Amide Derivative SA2 against Methicillin-Resistant *Staphylococcus aureus*

**DOI:** 10.3390/antibiotics11111497

**Published:** 2022-10-28

**Authors:** Moxi Yu, Yachen Hou, Meiling Cheng, Yongshen Liu, Caise Ling, Dongshen Zhai, Hui Zhao, Yaoyao Li, Yamiao Chen, Xiaoyan Xue, Xue Ma, Min Jia, Bin Wang, Pingan Wang, Mingkai Li

**Affiliations:** 1 Department of Pharmacology, School of Pharmacy, The Fourth Military Medical University, Xi’an 710032, China; 2 School of Pharmacy, Shaanxi University of Chinese Medicine, Xi’an 712046, China; 3 Department of Medicinal Chemistry and Pharmaceutical Analysis, School of Pharmacy, The Fourth Military Medical University, Xi’an 710032, China; 4 Department of Pharmacy, The Affiliated Hospital of Youjiang Medical University for Nationalities, Baise 533000, China

**Keywords:** *Staphylococcus aureus*, infection, squaric amide, alanine dehydrogenase

## Abstract

Methicillin-resistant *Staphylococcus aureus* (MRSA)-caused infection is difficult to treat because of its resistance to commonly used antibiotic, and poses a significant threat to public health. To develop new anti-bacterial agents to combat MRSA-induced infections, we synthesized novel squaric amide derivatives and evaluated their anti-bacterial activity by determining the minimum inhibitory concentration (MIC). Additionally, inhibitory activity of squaric amide 2 (SA2) was measured using the growth curve assay, time-kill assay, and an MRSA-induced skin infection animal model. A scanning electron microscope and transmission electron microscope were utilized to observe the effect of SA2 on the morphologies of MRSA. Transcriptome analysis and real-time PCR were used to test the possible anti-bacterial mechanism of SA2. The results showed that SA2 exerted bactericidal activity against a number of MRSA strains with an MIC at 4–8 µg/mL. It also inhibited the bacterial growth curve of MRSA strains in a dose-dependent manner, and reduced the colony formation unit in 4× MIC within 4–8 h. The infective lesion size and the bacterial number in the MRSA-induced infection tissue of mice were reduced significantly within 7 days after SA2 treatment. Moreover, SA2 disrupted the bacterial membrane and alanine dehydrogenase-dependent NAD^+^/NADH homeostasis. Our data indicates that SA2 is a possible lead compound for the development of new anti-bacterial agents against MRSA infection.

## 1. Introduction

*Staphylococcus aureus* (*S. aureus*) poses a serious threat to public health as it is the major cause for community- and hospital-acquired infections, including mild skin and soft tissue infections, septic arthritis, osteomyelitis, bacteremia, and lethal pneumonia [1]. The extensive use of antibiotics makes *S. aureus* a “superbug”, such as methicillin-resistant *S. aureus* (MRSA), which leads to persistent infections, antibiotic treatment failure, and poor clinical outcomes [2,3]. Recently, a number of reports and national surveillance data have identified MRSA as a significant pathogen, with an incidence ranging from 2.3% to 69.1% [4,5]. MRSA has become a major global healthcare problem and concern, and it is classified by the World Health Organization as one of 12 priority pathogens that threaten human health.

Increasing evidence shows that MRSA is not only resistant to β-lactams and aminoglycosides, but also to other traditional treatment agents such as quinolones and macrolides [6,7]. Although glycopeptide antibiotic vancomycin remains one of the last options for the treatment of severe MRSA infections, some *S. aureus* strains have started to exhibit increased resistance to vancomycin; these are known as vancomycin intermediate-resistant *S. aureus* (VISA) and vancomycin-resistant *S. aureus* [8,9]. In addition, with the introduction of linezolid, which is effective in the treatment of infections caused by various Gram-positive pathogens, including multi-drug-resistant *enterococci* and MRSA, resistance in *S. aureus* has also been encountered clinically [10]. Therefore, there is an urgent need to develop anti-bacterial agents with new chemical structures to combat drug-resistant *S. aureus*-induced infections.

Recently, small molecules with novel chemical structures were shown to exhibit potent anti-bacterial activity related to new targets or new mechanisms of action [11,12]. Squaric acid is a unique small molecule that possesses a symmetrical planar diprotic four-membered oxocarbon scaffold, which is widely used in bioorganic and medicinal chemistry. It has received considerable attention due to its molecular structure, which is capable of multiple interactions with biological targets [13]. In the last decades, a number of squaric acid derivatives were designed, and their pharmacological profiles were evaluated. Some of them, such as perzinfotel and navarixin, have entered various stages of clinical trials [14,15]. Interestingly, a series of glycopeptide antibiotics, including eremomycin, vancomycin, and ristocetin, which incorporate a squaric acid moiety, exert enhanced anti-bacterial activity against *Bacillus subtilis* and *Enterococcus faecalis* [16]. Interestingly, the squaric acid derivatives squaramides also exhibit activity against *Mycobacterium tuberculosis* [17]. However, the importance of squaric acid derivatives as privileged scaffolds with anti-bacterial activity is still largely unknown.

In this study, we synthesized and characterized squaric amides (SAs), novel squaric acid derivatives, and investigated their activity against common clinical pathogens in detail. Furthermore, the possible mechanism of anti-bacterial activity was also explored. This work highlights the potential of SAs as a novel anti-bacterial agent, especially against multi-drug-resistant bacteria.

## 2. Results

### 2.1. Chemistry

**Scheme 1:**
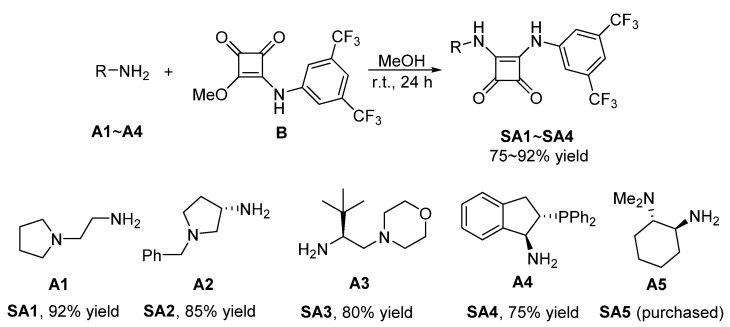
Synthetic route to squaric amide-derivative SAs.

To probe the possible anti-bacterial activity of SA derivatives, four novel SA compounds were synthesized and characterized, and one SA was purchased. The synthetic route to SAs is listed in Scheme 1. Four SAs were obtained from the coupling reactions of primary amines and mono-methyl ester B in good to excellent yields. This preparation process is quite simple and highly efficient. The characterization data and spectra copies of all synthetic SAs are presented in the Supplementary Information. The motifs of the primary amines in five SAs are different. SA1 contains an alkyl-linked pyrrolidine ring, and a chiral pyrrolidine ring is present in SA2. SA3 possesses a chiral diamino-group, which derived from tert-L-leucine, and SA4 was generated from a chiral indane-derived vicinal phophino-amine with the mono-methyl ester B. SA5 contains a chiral 1,2-diaminocyclohexane group. The structural diversity of SAs is beneficial to the bioassay. Two trifluoromethyl groups in all SAs may provide a good biocompatibility to the tested bacteria.

### 2.2. Screening and Validation of the SA Derivate with Potent Activity against MRSA

Firstly, we screened the possible anti-bacterial activity of SA derivatives against methicillin-susceptible *S. aureus* (ATCC 29213) and MRSA (300USA), and one of the characterized derivates termed SA2 showed the most potent activity against *S. aureus* strains (Supplementary Table S1). In additional, SA2 also showed potent activity against Gram-positive bacteria, including *S. aureus* clinical strains, *Staphylococcus epidermidis*, and *Enterococcus faecalis,* with MICs ranging from 2 µg/mL to 8 µg/mL, but had no obvious effect against Gram-negative bacteria, including *Escherichia coli*, *Salmonellosis typhimurium*, *Acinetobacter baumannii*, *Pseudomonas aeruginosa*, and *Klebsiella pneumoniae*. Interestingly, SA2 exerted bactericidal activity both against drug-susceptible Gram-positive bacteria and drug-resistant bacteria such as MRSA and VISA (Table 1).

### 2.3. Inhibitory Activity of SA2 to the Growth Curves and the Colony Formation Unit of MRSA

**Figure 1:**
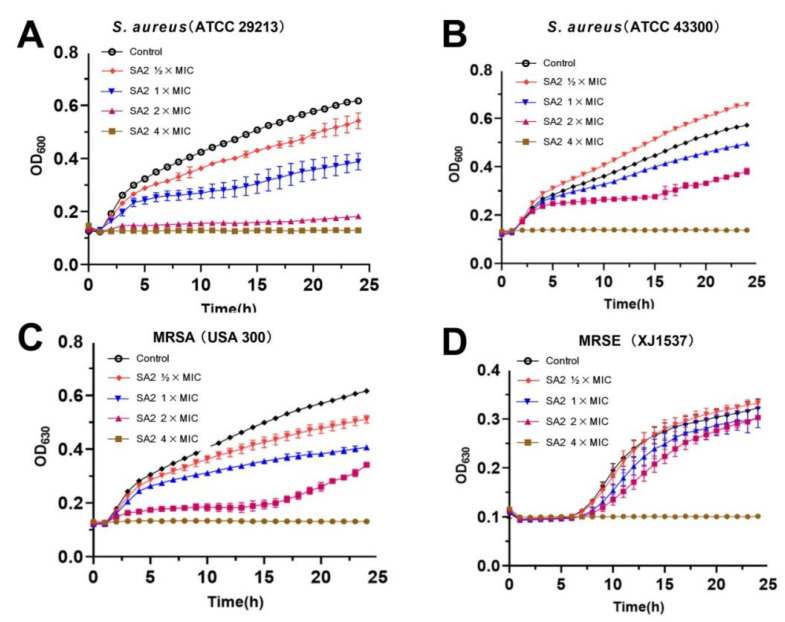
In vitro growth inhibition of SA2 to *S. aureus* and *S. epidermidis*. SA2 inhibited the growth curve of drug-susceptible *S. aureus* ATCC 29213 (**A**), *S. aureus* ATCC 43300 (**B**), MRSA USA300 (**C**), and methicillin-resistant *S. epidermidis* (MRSE) XJ1537 clinical isolated strain (**D**).

We also observed the growth inhibitory effect of SA2 against one methicillin-susceptible *S. aureus* (MSSA) strain (ATCC29213), two MRSA strains (ATCC43300 and USA300), and one MRSE clinical stain (XJ 537). The results showed that the growth of MSSA and MRSA strains were apparently inhibited by SA2 in 1×, 2×, and 4× MIC in a concentration-dependent manner (Figure 1).

**Figure 2:**
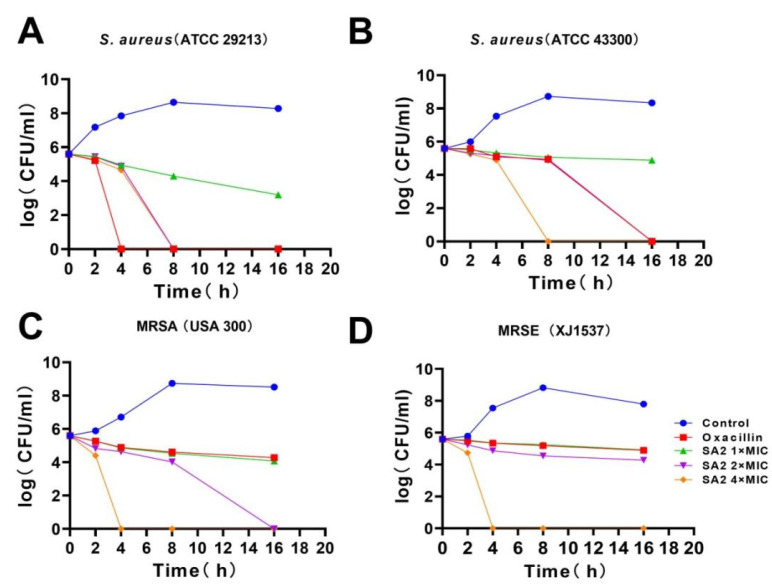
The bactericide of SA2 to *S. aureus* and *S. epidermidis*. The colony formation unit (CFU) of *S. aureus* ATCC 29213 (**A**), *S. aureus* ATCC 29213 (**B**), MRSA USA300 (**C**), and MRSE (XJ1537) (**D**) were decreased after SA2 treatment within 4–8 h at concentrations of 2× and 4× MIC.

In order to evaluate the pharmacodynamic function of SA2 against *S. aureus*, the relationship between the concentration of SA2 and the time point of bacterial growth was measured using the time-kill analysis. The results showed that SA2 could completely inhibit the colony formation unit (CFU) of MSSA strains (ATCC29213 and ATCC43300), MRSA strain (USA300), and VISA (Mu50) within 4–8 h at concentrations of 2× and 4× MIC. By contrast, the reference anti-bacterial agent oxacillin could not affect the survival of drug-resistant strains even in 4× MIC (Figure 2).

### 2.4. Effect of SA2 on the Morphologies of MRSA

**Figure 3:**
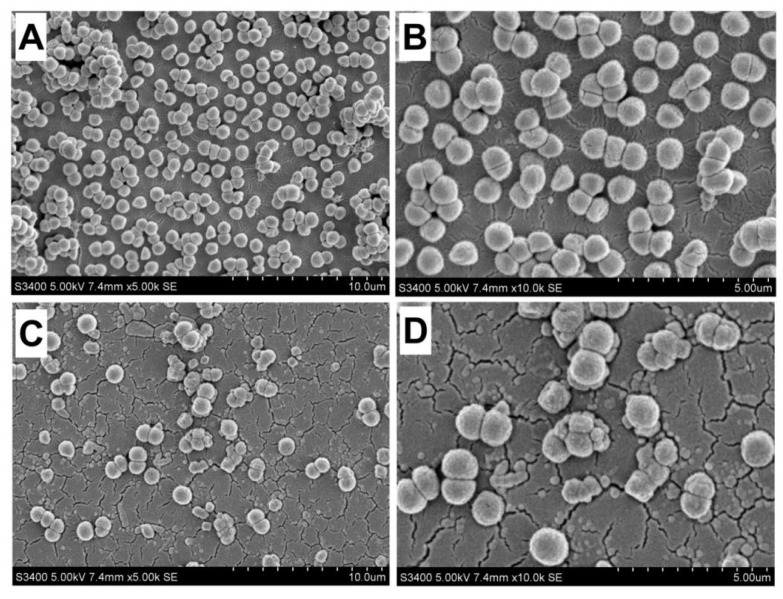
MRSA USA300 morphology investigated by scanning electron microscopy. (**A**,**B**) The control morphology of USA300 strain at 5000 and 10,000 times amplification of original magnification, respectively; (**C**,**D**) the morphology of USA300 strain treated by 4 μg/mL SA2 at 5000 and 10,000 times amplification of original magnification, respectively.

**Figure 4:**
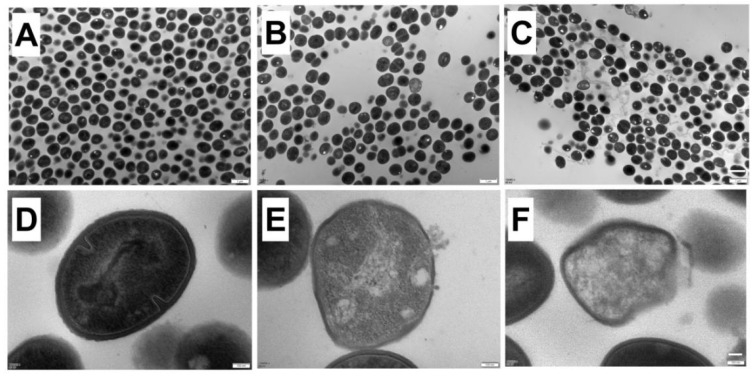
MRSA USA300 morphology investigated by transmission electron microscopy. (**A**–**C**) The morphology of USA300 strain in the control, 4 μg/mL SA2 treatment, and 8 μg/mL SA2 treatment group, respectively. The amplification of original magnification is 5000 times, Scale bar = 1 μm. (**D**–**F**) The morphology of USA300 strain in the control, 4 μg/mL, and 8 μg/mL SA2 treatment group, respectively. Scale bar = 100 nm. The amplification of original magnification is 50,000 times.

To further confirm the anti-bacterial activity of SA2, the morphologies of MRSA (USA300) were observed following 4 and 8 μg/mL of SA2 treatment under a scanning electron microscope (SEM) and transmission electron microscope (TEM). The results were consistent with those obtained by the growth curve assay and time-kill assay, and SA2 treatment induced a significant decrease in the number of bacteria. In addition, the SEM results showed that SA2 treatment caused the MRSA membrane to exhibit a more apparent coarse surface compared with the vehicle-treated control ones (Figure 3). Under TEM, more bacteria with broken membrane and cell debris were observed in the SA2 treatment (4 and 8 μg/mL) groups (Figure 4). These findings reinforced the bactericidal activity of SA derivatives and indicated that the disruption of the bacterial membrane may be involved in the anti-MRSA mechanism of SA2.

### 2.5. Therapeutic Efficacy of SA2 on MRSA-Induced Skin Infection

**Figure 5:**
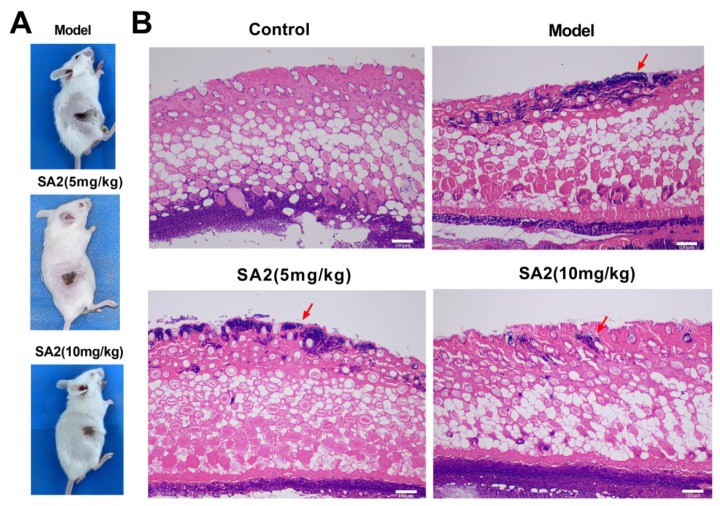
MRSA-induced skin infection was examined with H.E. staining in BALB/c mice. (**A**) The infective lesion size in the model control and intragastric administration of SA2 (5 mg/kg, 10 mg/kg) groups. (**B**) The pathological observation in the normal control, model control, and intragastric administration of SA2 (5 mg/kg, 10 mg/kg) groups. Arrows in red color indicate the inflammatory cells. Scale bar = 100 μm.

**Figure 6:**
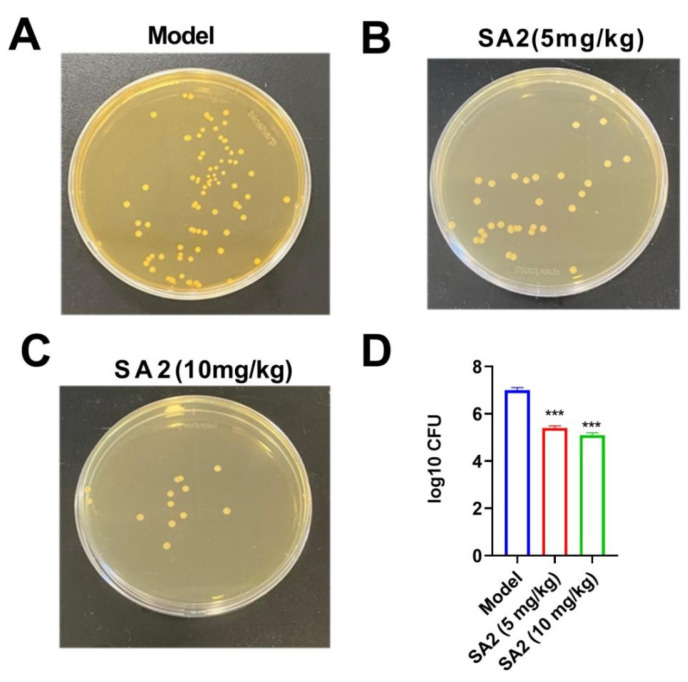
MRSA USA300 bacterial numbers in the infection tissue of mice were measured in the infection model control group (**A**), the 5 mg/kg SA2 treatment group (**B**), and the 10 mg/kg SA2 treatment group (**C**). The colony-forming units from the model control and SA treatment mice tissue were analyzed, *** *p* < 0.001 vs. model group, *n* = 6 (**D**).

Because *S. aureus* is a common cutaneous pathogen and is responsible for the great majority of bacterial skin infections [18], we evaluated the therapeutic effect of SA2 on mice with MRSA USA300-induced skin infection in vivo. Compared with the vehicle treatment control group, after daily intragastric administration of SA2 to mice at 5 mg/kg and 10 mg/kg of their body weight, the infected lesion size and the corresponding bacterial number in infected tissue of mice were reduced significantly within 7 days (Figure 5A and Figure 6). Moreover, the hematoxylin and eosin staining results revealed that SA2 treatment relieved pathological changes, including a thinner fat layer, decreased inflammatory cell infiltration, and less destruction of skin tissue structure compared with the vehicle-treated infection model group (Figure 5B).

### 2.6. SA2 Disrupted Alanine Dehydrogenase-Dependent NAD^+^/NADH Homeostasis

**Figure 7:**
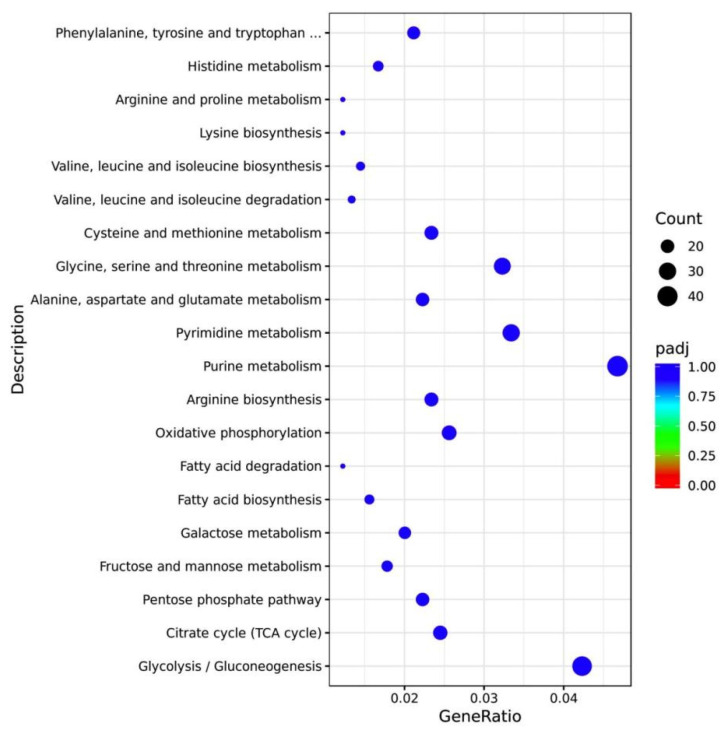
Transcriptome changes in MRSA USA300 after SA2 treatment; twenty COG enrichment differentially expressed genes were profiled.

To explore the possible anti-bacterial mechanisms of SA2, we performed transcriptome analysis on MRSA USA300 in the presence or absence of 8 µg/mL of compound SA2. The result revealed that SA2 regulated the expression of 209 genes (72 upregulated and 137 downregulated genes). The top 20 statistics of the pathway enrichment data showed that SA2 modulated the expression of genes mainly involved in biosynthesis and metabolic pathways, including the pyrimidine, purine, fatty acid, fructose and mannose, glycolysis, and citrate cycles (Figure 7).

**Figure 8:**
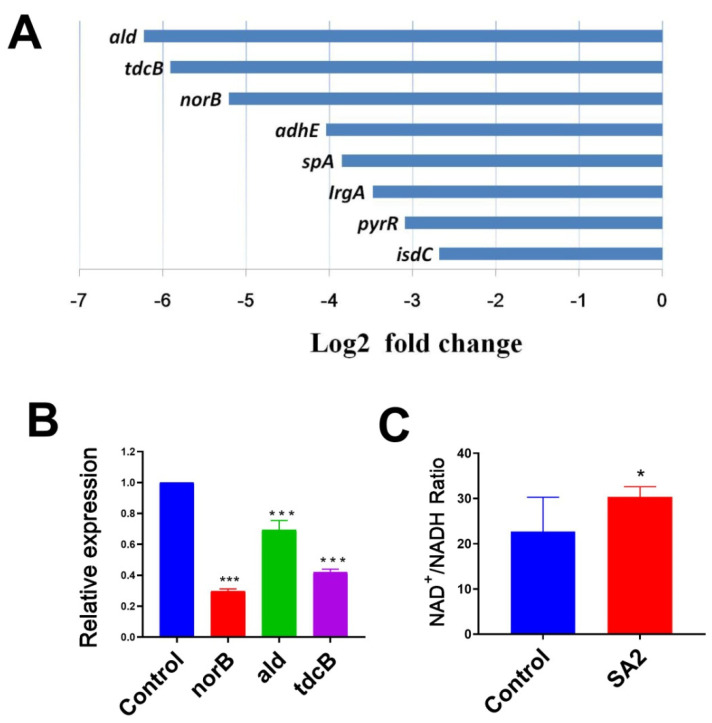
Profile of downregulated genes and NAD^+^/NADH ratio after SA2 treatment. The most significant log2fold changed gene in the transcriptomic profile (**A**). The expression of the *norB*, *ald,* and *tdcB* genes after SA2 treatment was measured by RT-PCR, *** *p* < 0.001 vs. model group, *n* = 3 (**B**). NAD^+^/NADH ratio was measured after SA2 treatment, * *p* < 0.05 vs. control group, *n* = 3 (**C**).

In the most differentially downregulated expressed genes, the log2fold change values of *ald*, *tdcB*, *norB*, *adhE*, *spA*, *lrgA*, *pyrR*, and *isdC* were −6.23, −5.92, −5.22, −4.05, −3.86, −3.48, −3.10, and −2.69, respectively (Figure 8A). These genes encode alanine dehydrogenase, pyridoxal-phosphate-dependent enzyme, multi-drug efflux MFS transporter, alcohol dehydrogenase, staphylococcal protein A, antiholin-like murein hydrolase modulator, pyr operon transcriptional regulator, and heme uptake protein, respectively. Next, the real-time PCR result confirmed that SA2 could significantly inhibit the expression of the *norB*, *ald*, and *tdcB* genes (Figure 8B).

Considering that *ald* was the most significantly changed gene in the transcriptomic profile, and alanine dehydrogenase encoded by *ald* catalyzes a reversible conversion of L-alanine to pyruvate with the concomitant oxidation of NADH to NAD^+^ in the micro-organisms [19], we measured the effect of SA2 on the NAD^+^/NADH ratio. Compared with the vehicle control, SA2 enhanced the NAD^+^/NADH ratio by about 1.5 times (Figure 8C). This result indicated that SA2 might exert anti-bacterial activity by disrupting bacterial alanine dehydrogenase-dependent NAD^+^/NADH homeostasis.

## 3. Discussion

To the best of our knowledge, this is the first report to focus on the anti-bacterial activity of SA. In this study, compound SA2 exhibited selective anti-bacterial activity against Gram-positive bacteria, including multi-drug-resistant *S. aureus*, without obvious activity against Gram-negative bacteria, such as *E. coli*, *P. aeruginosa*, *S. typhimurium*, *A. baumannii*, and *K. pneumoniae*. Although the underlying mechanism was not full uncovered, this might be related to the difference in the cellular structure of Gram-positive and Gram-negative bacteria. Moreover, the unique structure of the outer membrane of Gram-negative bacteria prevents the agent from entering into the cell, promotes anti-microbial resistance, and interprets bacterial signals from membrane-damaging agents [20,21].

The activity of antibiotics significantly relies on the chemical structure of small molecules, and the fluoro or trifluoromethyl substitute group usually plays a crucial role in improved anti-microbial activity [22,23]. Consistent with the structure–activity relationship, compound SA2 exhibited improved activity against drug-sensitive and -resistant *S. aureus* (MIC 2–8 μg/mL). It strengthened the importance of trifluoromethyl substitution in developing new potential therapeutics to combat multi-drug-resistant bacteria.

Metabolism supplies the biosynthetic intermediates necessary to the survival and pathogenesis of bacteria [24]. Our transcriptome analysis revealed that compound SA2 obviously altered the expression of the *ald* gene, which encodes alanine dehydrogenase, in *S. aureus*. Bacterial alanine dehydrogenase catalyzes the oxidative deamination of alanine to pyruvate and the reverse reaction, which is linked to the reduction/oxidation of NAD^+^/NADH and is crucial in the generation of energy through the tricarboxylic acid cycle in micro-organisms [19,25].

Our results also confirmed that compound SA2 disrupted the balance of NAD^+^/NADH, resulting in the generation of oxidative damage induced by the increase in the NAD^+^/NADH ratio, which is important in understanding the role of alanine dehydrogenase in bacterial metabolism. In *Mycobacterium smegmatis* and *M. tuberculosis*, the expression of the *ald* gene was regulated by the Lrp/AsnC family alanine dehydrogenase regulator (AldR) and strongly induced in the presence of alanine [26,27]. Although AldR is found in diverse bacterial species and alanine dehydrogenase is a potential drug target for treating tuberculosis [28,29], there is no evidence for the existence of AldR or the regulator with high homology to AldR in *S. aureus*. Thus, the regulatory mechanism of SA2 to *ald* expression remains to be elucidated.

SAs are remarkable four-membered ring systems derived from squaric acid, and their analogues have various biological activities that are enabled by the presence of significant H-bond donors and acceptors [30,31]. Hydrogen bonding and aromatic switching, in combination with structural rigidity, can mediate the cell wall disruption of a compound, which was observed under SEM and TEM. Considering that alanine dehydrogenase can catalyze the oxidative deamination of alanine to pyruvate [32], the downregulated activity of alanine dehydrogenase expression may also contribute to the bactericidal action of SA. Hence, an approach to identify novel SA derivatives that combat MRSA infection has important implications in developing effective anti-bacterial agents.

## 4. Materials and Methods

### 4.1. Bacterial Strains, Cells, and Chemicals

Bacterial strains including *S. aureus* (ATCC29213), *S. epidermidis* (ATCC14990), *Escherichia coli* (ATCC25922), and *Pseudomonas aeruginosa* (ATCC27853) were obtained from the Chinese National Centre for Surveillance of Antimicrobial Resistance. MRSA (XJ75302) and methicillin-resistant *S. epidermidis* (XJ75284) strains were isolated from the cultures of samples from patients in Xijing Hospital, the Fourth Military Medical University. MRSA (USA300) and MRSA (ATCC700699) strains were purchased from Microbiologics Company (Saint Cloud, MN, USA). All antibiotics were obtained from the National Institute for the Control of Pharmaceutical and Biological Products (Beijing, China). Five-week-old male BALB/c mice were from the Experimental Animal Center of the Fourth Military.

### 4.2. Synthesis and Characterization of SAs

The ^1^H NMR, ^13^C NMR, and ^19^F NMR spectra were measured in CDCl_3_ and MeOD solution on a Bruker AV-400 spectrometer using TMS as an internal reference. Coupling constant (*J*) values are given in Hz. Multiplicities are designated by the following abbreviations: s, singlet; d, doublet; t, triplet; q, quartet; br, broad; m, multiplet. High-resolution mass spectra (HRMS) were assessed on a Bruker microTOF-Q II Mass Spectrometer with ES ionization (ESI). All commercially available reagents were used as received. Thin-layer chromatography on silica (with GF_254_) was used to monitor all reactions. Products were purified by flash column chromatography on silica gel purchased from Qingdao Haiyang Chemical Co., Ltd. All solvents and organic and inorganic reagents were from commercial sources and used without purification unless otherwise noted. The primary amines A1~A4 and SA5 were purchased from Daicel chiral technologies (China) Co. Ltd. The mono-methyl ester B was prepared according to the methods reported in the literature [33]. SA1, SA2, and SA3 are known compounds [34,35,36]. The characterization data of all new compounds are listed in the Supplementary Information.

The preparation of mono-methyl ester B [33]. The mixture of 3,4-dimethoxy-3-cyclobutene-1,2-dione (1.00 g, 7 mmol) and 3,5-bis(trifluoromethyl)aniline (1.20 mL, 7.7 mmol, 1.1 equiv) in MeOH (10 mL) was stirred at r.t. for 2d, producing a yellow precipitate. The yellow precipitate was filtered with the aid of cold MeOH to provide a yellow powder, which was dried in vacuo to (2.15 g, 90%). The ^1^H NMR and ^13^C NMR spectra were identical to those reported in the literature.

General procedure for the preparation of squaramides (SA1-SA4) [35,36,37]. A solution of corresponding amine (3.0 mmol) in MeOH (5 mL) was added to a solution of mono-metyl ester (B, 1.02 g, 3.0 mmol) in MeOH (15 mL) at room temperature (r.t.). The mixture was stirred for 24 h. The reaction mixture was filtered, and the precipitate was washed with cold MeOH (2 × 5.0 mL) to afford pure squaramides SA1-SA4, respectively. They were analytically pure and used in the in vitro and in vivo experiments without further purification.

SA1: white powder, 92% yield; m.p. 145.2–146.1 °C; ^1^H NMR (400 MHz, DMSO-*d*_6_) δ: 10.31 (s, 1H), 8.06 (s, 2H), 7.80 (s, 1H), 7.66 (s, 1H), 3.75 (s, 2H), 3.35 (4H, merged), 2.65 (s, 2H), 2.51 (4H, merged); ^13^C NMR (101 MHz, DMSO-*d*_6_) δ 185.4, 178.5, 162.3, 141.2, 131.5, 124.5, 121.8, 117.9, 114.6, 55.7, 53.5, 42.8, 23.1; ^19^F NMR (376 MHz, DMSO-*d*_6_) δ: −61.8; HRMS (ESI) *m*/*z* calcd. for C_18_H_18_F_6_N_3_O_2_^+^ [M+H]^+^: 422.1298, found 422.1302.

SA2: light yellow powder, 85% yield; m.p. 167.1–168.5 °C; [α]${}_{D}^{20}$ + 63.7° (c = 0.25, CH_2_Cl_2_); ^1^H NMR (400 MHz, MeOD) δ: 8.05 (bs, 2H), 7.55 (bs, 1H), 7.34–7.26 (m, 5H), 4.78 (s, 1H), 3.70 (s, 1H), 3.33 (s, 1H), 2.94 (br, 1H), 2.77 (bs, 2H), 2.45 (bs, 2H), 1.84 (bs, 1H), 4.73 (q, *J* = 6.9 Hz, 1H), 4.38–4.26 (m, 2H), 3.82 (dd, *J* = 12.3, 6.8 Hz, 1H), 2.86 (t, *J* = 7.0 Hz, 1H), 2.71 (s, 3H), 1.95 (bs, 1H), 1.52 (s, 3H), 1.35 (s, 3H); ^13^C NMR (100 MHz, MeOD) δ: 169.0, 162.6, 140.9, 132.7, 128.8, 128.1, 127.1, 124.5, 121.8, 117.9, 115.2, 60.5, 59.5, 53.8, 52.2, 32.8; ^19^F NMR (376 MHz, MeOD) δ: −64.5; HRMS (ESI) *m*/*z* calcd. for C_23_H_20_F_6_N_3_O_2_^+^ [M+H]^+^: 484.1454, found 484.1453.

SA3: white powder, 80% yield; m.p. > 230 °C; [α]${}_{D}^{20}$ + 5.3° (c = 0.22, CH_2_Cl_2_); ^1^H NMR (400 MHz, MeOD) δ: 8.14 (s, 2H), 7.59 (s, 1H), 4.18 (d, *J* = 10.28 Hz, 1H), 3.62 (br, 4H), 2.70–2.65 (m, 3H), 2.48 (t, *J* = 12.0 Hz, 1H), 2.37 (br, 2H), 1.05 (s, 9H); ^13^C NMR (100 MHz, MeOD) δ: 184.3, 180.5, 170.7, 161.9, 140.8, 132.8 (d, *J*_C-F_ = 33 Hz), 124.5, 121.8, 117.8, 115.5, 66.8, 60.4, 59.5, 53.9. 33.7, 25.7; ^19^F NMR (376 MHz, MeOD) δ: −64.0; HRMS (ESI) *m*/*z* calcd. for C_22_H_26_F_6_N_3_O_3_^+^ [M+H]^+^: 494.1873, found 494.1886.

SA4: white powder, 75% yield; m.p. 141.9–143.1°C; [α]${}_{D}^{20}$ + 149.4° (c = 0.05, CH_2_Cl_2_); ^1^H NMR (400 MHz, Chloroform-*d*) δ: 9.79 (s, 1H), 8.04 (s, 2H), 7.82 (d, *J* = 6.9 Hz, 2H), 7.74 (d, *J* = 9.9 Hz, 1H), 7.58 (s, 1H), 7.53 (t, *J* = 7.7 Hz, 2H), 7.37–7.30 (m, 1H), 7.24 (t, *J* = 7.5 Hz, 2H), 7.17 (s, 2H), 7.03–6.86 (m, 2H), 6.88–6.81 (m, 2H), 5.65–5.40 (m, 1H), 3.62 (s, 1H), 2.81 (s, 1H), 2.72–2.51 (m, 1H); ^13^C NMR (101 MHz, Chloroform-*d*) δ: 184.7, 178.9, 167.8, 163.7, 141.0 (dd, *J* = 29.3, 8.08 Hz), 139.4, 136.7 (d, *J* = 12.5 Hz), 135.3 (dd, *J* = 32.3, 12.1 Hz), 132.8 (d, *J* = 20.2 Hz), 132.7 (q, *J* = 33.3 Hz), 129.3 (d, *J* = 33.3 Hz), 128.7 (d, *J* = 7.4 Hz), 128.5 (t, *J* = 8.1 Hz), 126.9, 124.4, 123.7, 123.0 (q, *J*C-F = 272 Hz), 122.9, 119.3, 117.1, 64.5 (d, *J* = 26.8 Hz), 44.6 (d, *J* = 10.7 Hz), 35.4 (d, *J* = 21.5 Hz); ^19^F NMR (376 MHz, Chloroform-*d*) δ: −63.1; ^31^P NMR (162 MHz, Chloroform-*d*) δ: −8.4; HRMS (ESI) *m*/*z* calcd. for C_33_H_24_F_6_N_2_O_2_P^+^ [M+H]^+^: 625.1469, found: 625.1470.

SA5 was purchased from Daicel Chiral Technologies (China) Co., Ltd. for use in the lab; HRMS (ESI) *m*/*z* calcd. for C_22_H_24_F_6_N_3_O_2_^+^ [M+H]^+^: 476.1767, found: 476.1767.

### 4.3. Determination of Minimal Inhibitory Concentration (MIC)

The MIC was determined based on the Clinical and Laboratory Standards Institute broth micro-dilution method, as previously reported [37]. In brief, bacterial strains were grown overnight in Mueller–Hinton (MH) broth. A total of 100 μL of bacterial suspensions containing 5 × 10^5^ CFU/mL was added to each well of a sterile 96-well micro-titer plate. Subsequently, 100 μL of each test compound diluted in the phosphate buffer saline (PBS) was added to the micro-plates followed by incubation at 37 °C for 24 h. The final concentration ranged from 0.5 μg/mL to 256 μg/mL. About 50 µL of 0.2% triphenyl tetrazolium chloride (TTC) was added to each well of micro-titer plates and incubated at 37 °C for 1.5 h. The TTC-based MIC was determined as the lowest concentration of the derivatives that showed no red color change.

### 4.4. Bacterial Growth Curve Assay

The effect of SA on the growth curve of *S. aureus* (ATCC 29213), *S. aureus* (ATCC 43300), MRSA (USA300), and MRSE (XJ1537) was measured as follows: The triplicate bacterial cells were diluted to 5 × 10^5^ CFU/mL, and then different concentrations (1/2× MIC, 1× MIC, 2× MIC and 4× MIC) of compound SA2 in MH broth medium (150 µL) were added into the 150 µL cell suspension and incubated at 37 °C in the automated Bioscreen C system (Labsystems, Helsinki, Finland). The density of the bacterial cell suspensions was measured at 600 nm at 10 min intervals for 48 h. The control was added with MH broth medium without the test compound.

### 4.5. Time-Kill Assay

The compound was incorporated into 10 mL of MH broth at 1× MIC, 2× MIC, and 4× MIC. Bacteria inoculated in MH broth without compound or antibiotic were set as the negative control group, and oxacillin treatment was used as the positive control group. After incubating at 37 °C, emergent bacterial colonies were counted at 2, 4, 8, 16, and 24 h time points. The bacterial colonies were recorded, and the counts of different treatments were compared.

### 4.6. MRSA-Induced Skin Infection Animal Model

BALB/c mice were anesthetized via intraperitoneal injection of 2.5 mL/kg of 10% chloral hydrate. A rectangular area of approximately 2 × 3 cm was shaved off on the lower back of mice, and the tissue was disinfected with 75% alcohol. Suspensions containing MRSA (USA300) at 1 × 10^8^ CFU/mL was injected into the subcutaneous tissue of mice. After 24 h, 5 or 10 μg/mL of compound SA2 was intraperitoneally injected for six consecutive days. Then, the infection area size, pathological change, and bacterial burden were measured.

### 4.7. Electron Microscopy Observation

MRSA (USA300) at 1 × 10^8^ CFU/mL was cultured in MH broth with or without compound at different concentrations (4, 8, and 24 μg/mL). Then, the bacterial cells were harvested and washed with 0.01 M PBS twice, and added to 3% glutaraldehyde for microtome section. The samples were post-fixed by 1% osmium tetroxide (OsO_4_) and dehydrated in 50%, 70%, 80%, 90%, and 95% acetone for 15 min, successively. The images of the sample were observed and recorded under a scanning electron microscope (HITACHI S-3400N, Hitachi, Tokyo, Japan) or transmission electron microscope (JEM-1230, JOEL, Tokyo, Japan).

### 4.8. Transcriptome Analysis

The total RNA of control and SA2-treated MRSA (USA300) samples were extracted with Trizol reagent, and the cleaved RNA fragments were reverse transcribed to generate the sequencing libraries using a gene sequencing system (HiSeq 2000, Illumina, San Diego, CA, USA). The expression of genes between the control groups and SA2 treatment groups were measured and analyzed as follows: the RNA-seq data were normalized and log-transformed using an oligo R package and multi-array average method, respectively. The significant differences in gene expression were analyzed as Log2 (fold change) ≥ 1 and a *Padj* value ≤ 0.05.

### 4.9. Real-Time PCR

The total RNA from the MRSA (USA300) strain was isolated using the RNeasy kit (QIAGEN, Shanghai, China), and the sequences of primers are listed in Supplementary Table S2. The isolated RNA was quantified and reverse-transcribed into cDNA using PrimeScript RT reagent kit, and then RT-PCR was performed using Premix Taq RT-PCR System (Takara Bio Inc., Kyoto, Japan) as follows: cDNA was denatured at 95 °C for 30 s, amplified by 40 cycles of 95 °C for 10 s, 60 °C for 31 s, 95 °C for 15 s, 60 °C for 1min, and 95 °C for 15 s. The data were analyzed using the 2^−∆∆Ct^ method compared to control group.

### 4.10. Statistical Analysis

The results are expressed as mean ± SD. One-way analysis of variance (ANOVA) and two-way ANOVA followed by Dunnett’s *t*-test were used for statistical evaluations, with a *p* < 0.05 indicative of statistical significance.

## 5. Conclusions

SA derivatives were synthesized and characterized in the present study. The anti-bacterial activity of these compounds against Gram-positive and Gram-negative bacteria was evaluated, and compound SA2 was shown to inhibit drug-susceptible *S. aureus* and MRSA in vitro and in vivo. SA possibly exerts its anti-bacterial activity through the downregulation of alanine dehydrogenase, which encodes gene expression and NAD^+^/NADH balance disruption.

## Figures and Tables

**Table 1:** Activity of SA2 against pathogenic bacteria.

Microorganism	MIC (µg/mL)
*Staphylococcus aureus* (MSSA, ATCC 29213)	2–4
*Staphylococcus aureus* (MSSA, ATCC43300)	4
*Staphylococcus aureus* (MRSA, VISA, Mu50)	4
*Staphylococcus aureus* (MRSA, USA300)	4
*Staphylococcus aureus* (MRSA, clinical strain XJ 26)	8
*Staphylococcus aureus* (MRSA, clinical strain XJ 216)	8
*Staphylococcus aureus* (MRSA, clinical strain XJ 317)	8
*Staphylococcus aureus* (MRSA, clinical strain XJ 141240)	8
*Staphylococcus epidermidis* (ATCC14990)	8
*Staphylococcus epidermidis* (MRSE, clinical strain XJ 537)	4
*Enterococcus faecalis* (ATCC29212)	4
*Enterococcus faecalis* (VER clinical strain XJ 21)	8
*Enterococcus faecalis* (VER clinical strain XJ 22)	8
*Enterococcus faecalis* (VER clinical strain XJ 23)	8
*Escherichia coli* (ATCC 25922)	>256
*Escherichia coli* (MDR, clinical strain XJ 74283)	>256
*Salmonella typhimurium* (MDR, SL1344)	>256
*Acinetobacter baumannii* (ATCC 19606)	>256
*Pseudomonas aeruginosa* (ATCC 27853)	>256
*Pseudomonas aeruginosa* (MDR, clinical strain XJ 75315)	>256
*Klebsiella pneumoniae* (ATCC700603)	>256
*Klebsiella pneumoniae* (ATCC13885)	>256
*Klebsiella pneumoniae* (MDR, ATCC 75297)	>256

## Data Availability

Data are contained within the article and Supplementary Materials.

## Supplementary Materials

The following supporting information can be downloaded at: https://www.mdpi.com/article/10.3390/antibiotics11111497/s1, Supplementary Figure: Characterization of SA compounds; Supplementary Table S1: Anti-bacterial activity of SA compounds; Supplementary Table S2: Primers of RT-PCR.
