# Supplementary material for: Antibacterial Activity of Squaric Amide Derivative SA2 against Methicillin-Resistant Staphylococcus aureus
Source: Antibiotics (Basel). 2022 Oct 28;11(11):1497. doi: 10.3390/antibiotics11111497 (PMC9686510; doi:10.3390/antibiotics11111497)
Supplement: Supplementary file 1 [file antibiotics-11-01497-s001.zip › antibiotics-1935192-supplementary.pdf]

antibiotics

MDPI

# Supplementary Information

## General

The $^{1}H$ NMR, $^{13}C$ NMR, $^{19}F$ NMR spectra were measured in $CDCl_3$ and MeOD solution on a Bruker AV-400 spectrometer using TMS as an internal reference. Coupling constant (*J*) values are given in Hz. Multiplicities are designated by the following abbreviations: s, singlet; d, doublet; t, triplet; q, quartet; br, broad; m, multiplet. High-resolution mass spectra (HRMS) were performed on a Bruker microTOF-Q II Mass Spectrometer with ES ionization (ESI). All commercially available reagents were used as received. Thin-layer chromatography on silica (with $GF_{254}$) was used to monitor all reactions. Products were purified by flash column chromatography on silica gel purchased from Qingdao Haiyang Chemical Co., Ltd. All solvents, organic and inorganic reagents were from commercial sources and used without purification unless otherwise noted. The primary aminess **A1~A4** and **SA5** are purchased from Daicel chiral technologies (China) Co. Ltd. The mono-methyl ester **B** was prepared according to literatures reported method.[1] **SA1**, **SA2** and **SA3** are known compounds.[2-4] The characterization data of all new compounds were listed in the Supporting Information.

**The preparation of mono-methyl ester B**[1]

The mixture of 3,4-dimethoxy-3-cyclobutene-1,2-dione (1.00 g, 7 mmol) and 3,5-bis(trifluoromethyl)aniline (1.20 mL, 7.7 mmol, 1.1 equiv) in MeOH (10 mL) was stirred at r.t. for 2d to give yellow precipitate. The yellow precipitate was filtered with the aid of cold MeOH to provide a yellow powder and dried in vacuo to (2.15 g, 90%).

**mono-methyl ester B**: $^{1}H$ NMR (400 MHz, MeOD) δ: 8.05 (s, 2H), 7.68 (s, 1H), 4.51 (s, 3H, $OCH_3$).

**General procedure for preparation of squaramides (SA1-SA4)** [2-4]

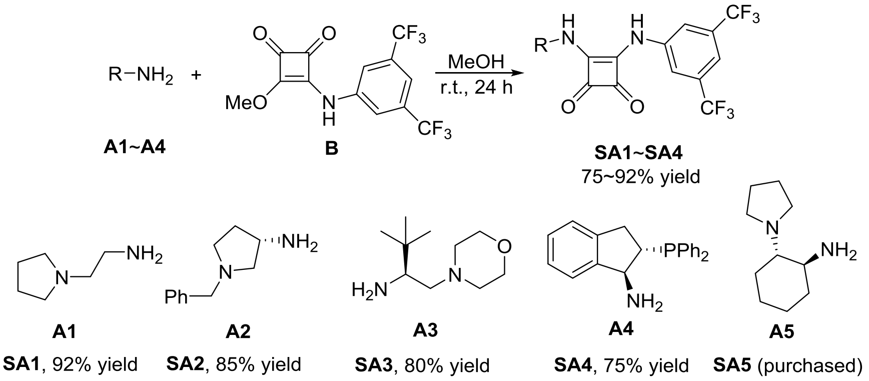


To a solution of mono-metyl ester (**B**, 1.02 g, 3.0 mmol) in MeOH (15 mL) was added a solution of corresponding amine (3.0 mmol) in MeOH (5 mL) at room temperature (r.t.). The mixture was stirred for 24 h. The reaction mixture was filtered, and the precipitate was washed with cold MeOH (2 × 5.0 mL) to afford pure squaramides **SA1-SA4**, respectively. They are analytical pure and used in the *vitro* and *vivo* experiments without further purification.

SA1: white powder, 92% yield; m.p. 145.2-146.1 °C; $^{1}H$ NMR (400 MHz, DMSO-$d_6$) δ: 10.31 (s, 1H), 8.06 (s, 2H), 7.80 (s, 1H), 7.66 (s, 1H), 3.75 (s, 2H), 3.35 (4H, merged), 2.65 (s, 2H), 2.51 (4H, merged); $^{13}C$ NMR (101 MHz, DMSO-$d_6$) δ 185.4, 178.5, 162.3, 141.2, 131.5, 124.5, 121.8, 117.9, 114.6, 55.7, 53.5, 42.8, 23.1; $^{19}F$ NMR (376 MHz, DMSO-$d_6$) δ: -61.8; HRMS (ESI) *m/z* calcd. for $C_{18}H_{18}F_6N_3O_2^+$ $[M+H]^+$: 422.1298, found 422.1302.

SA2: light yellow powder, 85% yield; m.p. 167.1-168.5 °C; [*α*] $^{20}_{D}$ +63.7° (c = 0.25, $CH_2Cl_2$); $^1$H NMR (400 MHz, MeOD) δ: 8.05 (bs, 2H), 7.55 (bs, 1H), 7.34-7.26 (m, 5H), 4.78 (s, 1H), 3.70 (s, 1H), 3.33 (s, 1H), 2.94 (br, 1H), 2.77 (bs, 2H), 2.45 (bs, 2H), 1.84 (bs, 1H), 4.73 (q, *J* = 6.9 Hz, 1H), 4.38-4.26 (m, 2H), 3.82 (dd, *J* = 12.3, 6.8 Hz, 1H), 2.86 (t, *J* = 7.0 Hz, 1H), 2.71 (s, 3H), 1.95 (bs, 1H), 1.52 (s, 3H), 1.35 (s, 3H); $^{13}$C NMR (100 MHz, MeOD) δ: 169.0, 162.6, 140.9, 132.7, 128.8, 128.1, 127.1, 124.5, 121.8, 117.9, 115.2, 60.5, 59.5, 53.8, 52.2, 32.8; $^{19}$F NMR (376 MHz, MeOD) δ: -64.5; HRMS (ESI) *m/z* calcd. for $C_{23}H_{20}F_6N_3O_2^+$ [M+H]$^+$: 484.1454, found 484.1453.

SA3: white powder, 80% yield; m.p. > 230 °C; [*α*] $^{20}_{D}$ +5.3° (c = 0.22, $CH_2Cl_2$); $^1$H NMR (400 MHz, MeOD) δ: 8.14 (s, 2H), 7.59 (s, 1H), 4.18 (d, J = 10.28 Hz, 1H), 3.62 (br, 4H), 2.70-2.65 (m, 3H), 2.48 (t, *J* = 12.0 Hz, 1H), 2.37 (br, 2H), 1.05 (s, 9H); $^{13}$C NMR (100 MHz, MeOD) δ: 184.3, 180.5, 170.7, 161.9, 140.8, 132.8 (d, $J_{C\text{-}F}$ = 33 Hz), 124.5, 121.8, 117.8, 115.5, 66.8, 60.4, 59.5, 53.9. 33.7, 25.7; $^{19}$F NMR (376 MHz, MeOD) δ: -64.0; HRMS (ESI) *m/z* calcd. for $C_{22}H_{26}F_6N_3O_3^+$ [M+H]$^+$: 494.1873, found 494.1886.

SA4: white powder，75% yield; m.p. 141.9- 143.1°C; [*α*] $^{20}_{D}$ +149.4° (c = 0.05, $CH_2Cl_2$); $^1$H NMR (400 MHz, Chloroform-*d*) δ: 9.79 (s, 1H), 8.04 (s, 2H), 7.82 (d, *J* = 6.9 Hz, 2H), 7.74 (d, *J* = 9.9 Hz, 1H), 7.58 (s, 1H), 7.53 (t, *J* = 7.7 Hz, 2H), 7.37-7.30 (m, 1H), 7.24 (t, *J* = 7.5 Hz, 2H), 7.17 (s, 2H), 7.03-6.86 (m, 2H), 6.88-6.81 (m, 2H), 5.65-5.40 (m, 1H), 3.62 (s, 1H), 2.81 (s, 1H), 2.72-2.51 (m, 1H); $^{13}$C NMR (101 MHz, Chloroform-*d*) δ: 184.7, 178.9, 167.8, 163.7, 141.0 (dd, *J* = 29.3, 8.08 Hz), 139.4, 136.7 (d, *J* = 12.5 Hz), 135.3 (dd, *J* = 32.3, 12.1 Hz), 132.8 (d, *J* = 20.2 Hz), 132.7 (q, *J* = 33.3 Hz), 129.3 (d, *J* = 33.3 Hz), 128.7 (d, *J* = 7.4 Hz), 128.5 (t, *J* = 8.1 Hz), 126.9, 124.4, 123.7, 123.0 (q, *J*C-F = 272 Hz), 122.9, 119.3, 117.1, 64.5 (d, *J* = 26.8 Hz), 44.6 (d, *J* = 10.7 Hz), 35.4 (d, *J* = 21.5 Hz); $^{19}$F NMR (376 MHz, Chloroform-*d*) δ: -63.1; $^{31}$P NMR (162 MHz, Chloroform-*d*) δ: -8.4; HRMS (ESI) *m/z* Calculated for $C_{33}H_{24}F_6N_2O_2P^+$ [M+H]$^+$: 625.1469, found: 625.1470.

SA5 was purchased from Daicel Chiral Technologies (China) Co., Ltd. for only used in Lab; HRMS (ESI) *m/z* Calculated for $C_{22}H_{24}F_6N_3O_2^+$ [M+H]$^+$: 476.1767, found: 476.1767.

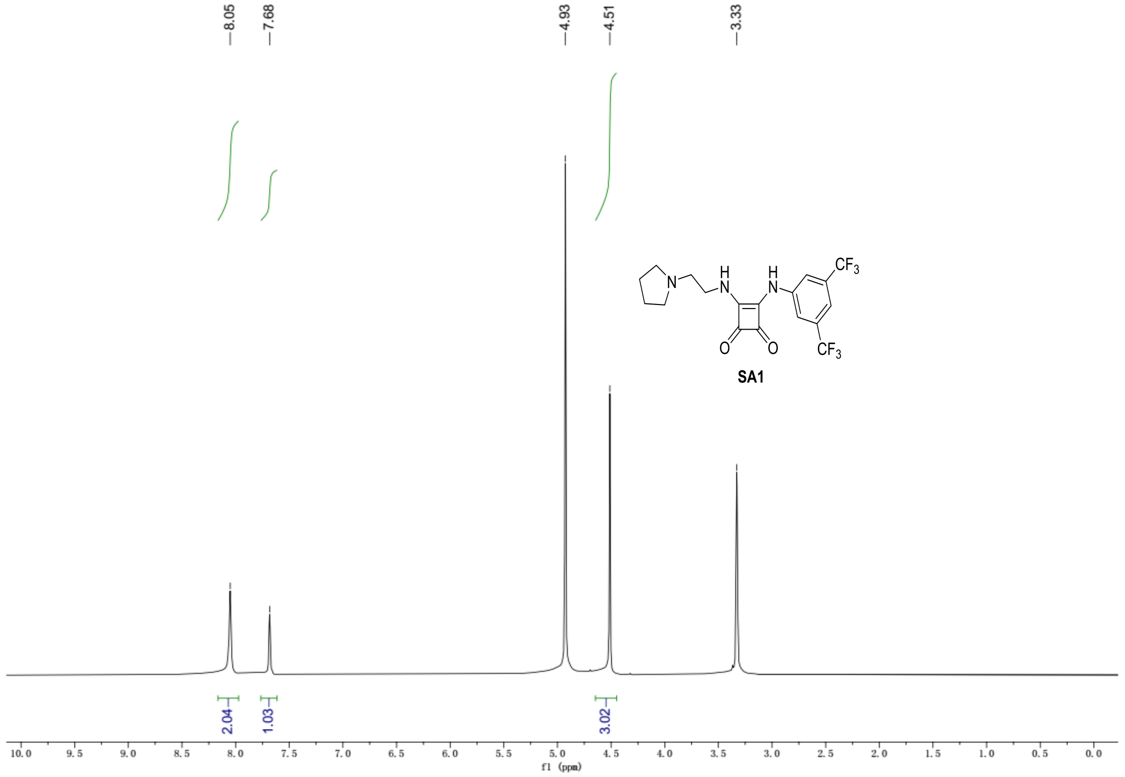


$^1$H NMR for **B**

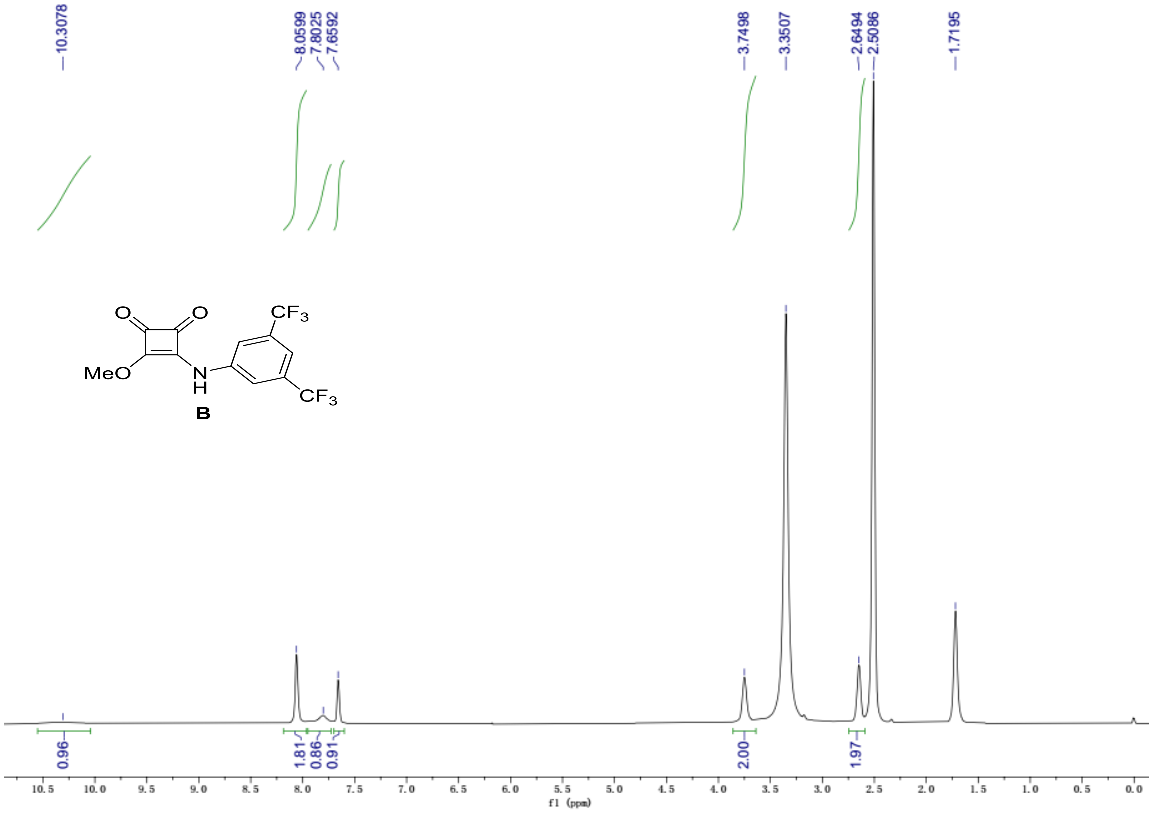


$^1$H NMR for **SA1**

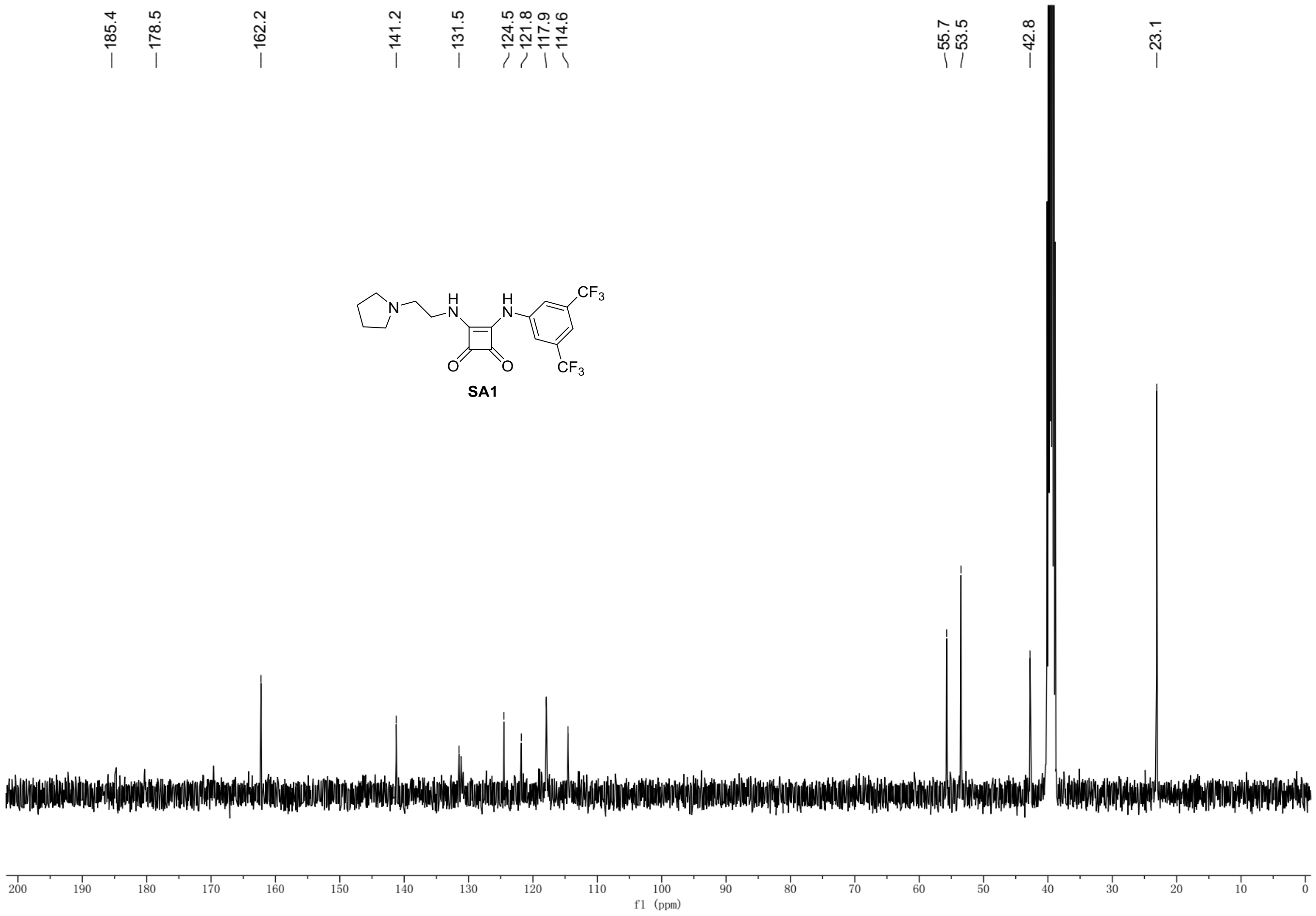


$^{13}$C NMR for **SA1**

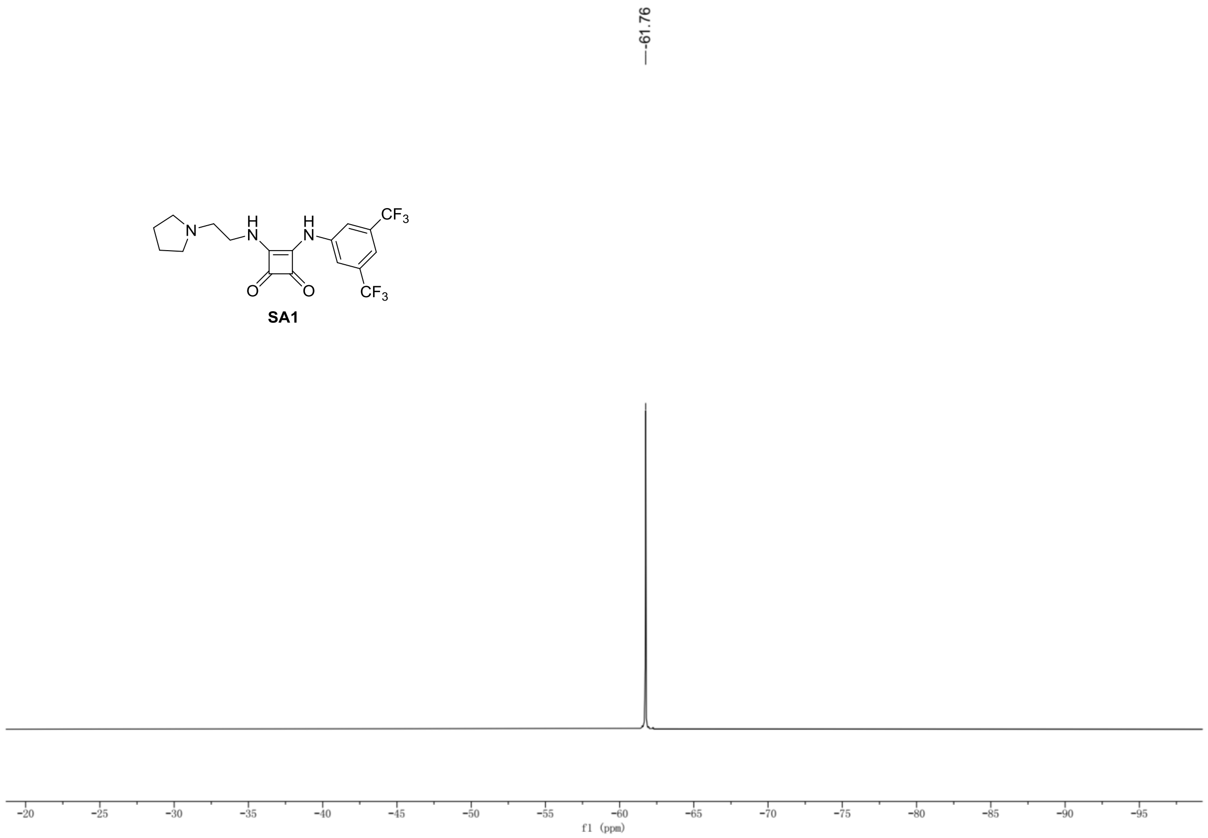


$^{19}$F NMR for **SA1**

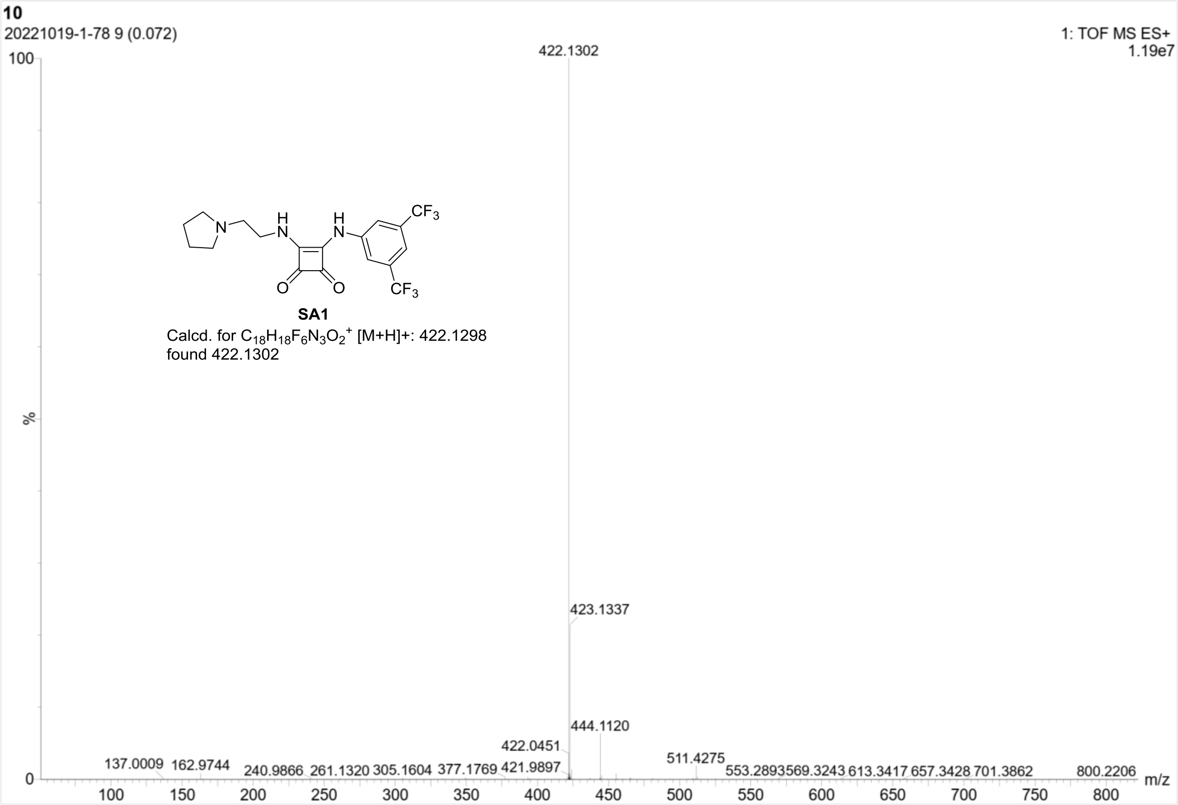
10
20221019-1-78 9 (0.072)
1: TOF MS ES+
1.19e7
422.1302
SA1
Calcd. for C18H18F6N3O2+ [M+H]+: 422.1298
found 422.1302
423.1337
444.1120
422.0451
421.9897
137.0009
162.9744
240.9866
261.1320
305.1604
377.1769
511.4275
553.2893
569.3243
613.3417
657.3428
701.3862
800.2206
%
m/z

HRMS for **SA1**

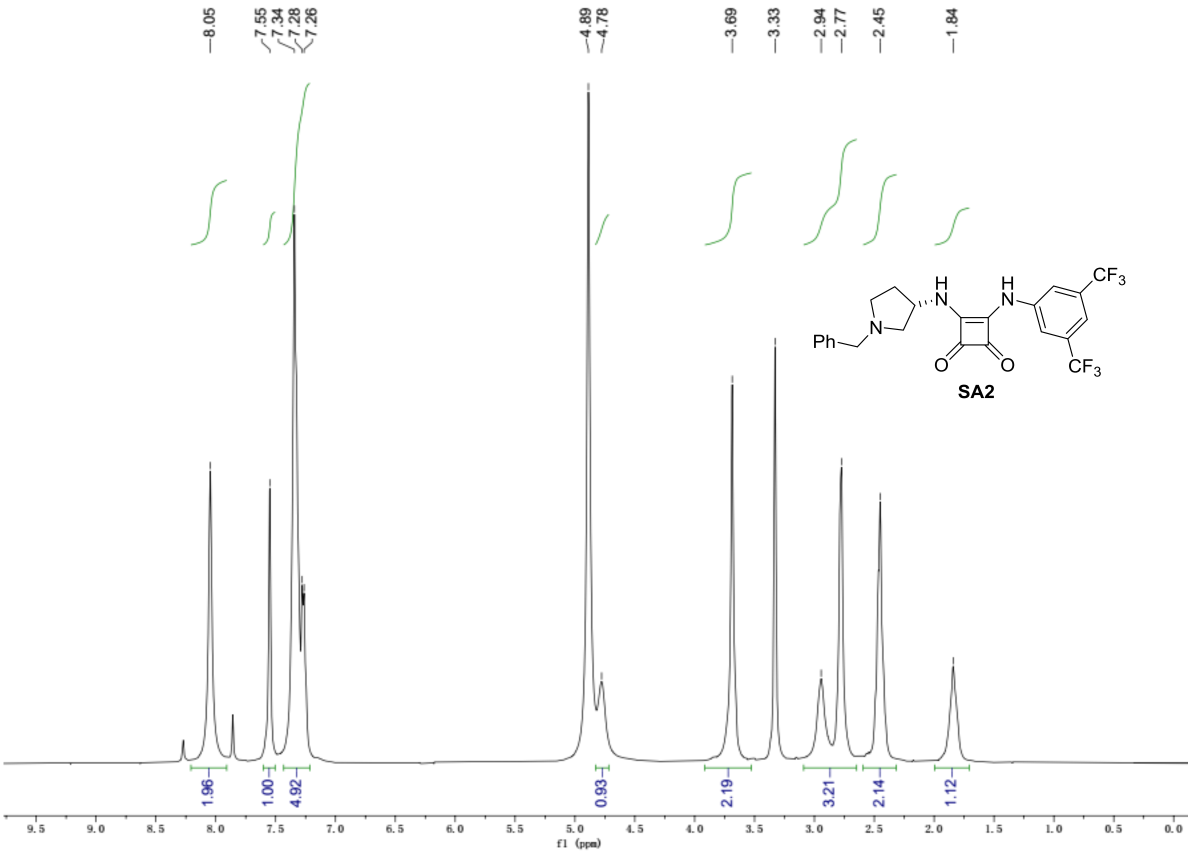
8.05
7.55
7.34
7.28
7.26
4.89
4.78
3.69
3.33
2.94
2.77
2.45
1.84
1.96
1.00
4.92
0.93
2.19
3.21
2.14
1.12
9.5
9.0
8.5
8.0
7.5
7.0
6.5
6.0
5.5
5.0
4.5
4.0
3.5
3.0
2.5
2.0
1.5
1.0
0.5
0.0
f1 (ppm)
Ph
N
H
N
H
N
CF3
CF3
O
O
SA2

$^1$H NMR of **SA2**

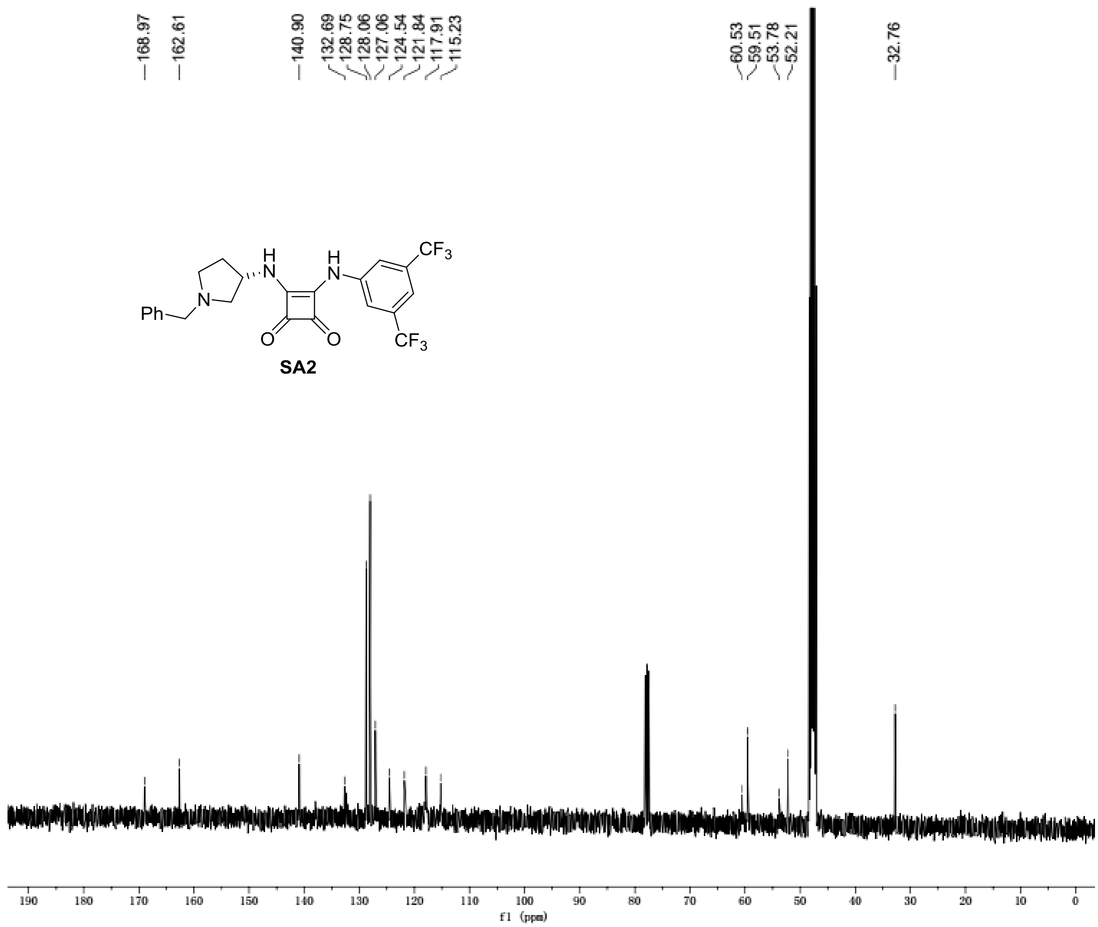


$^{13}$C NMR of **SA2**

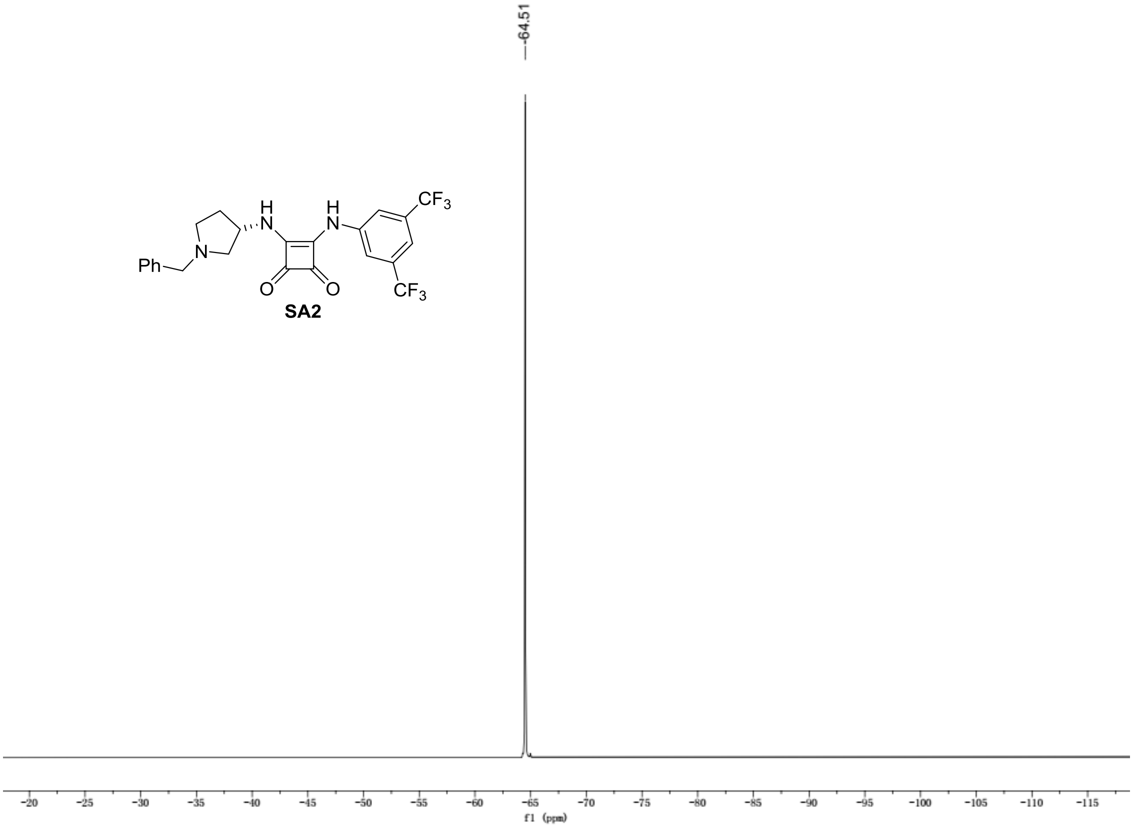


$^{19}$F NMR of **SA2**

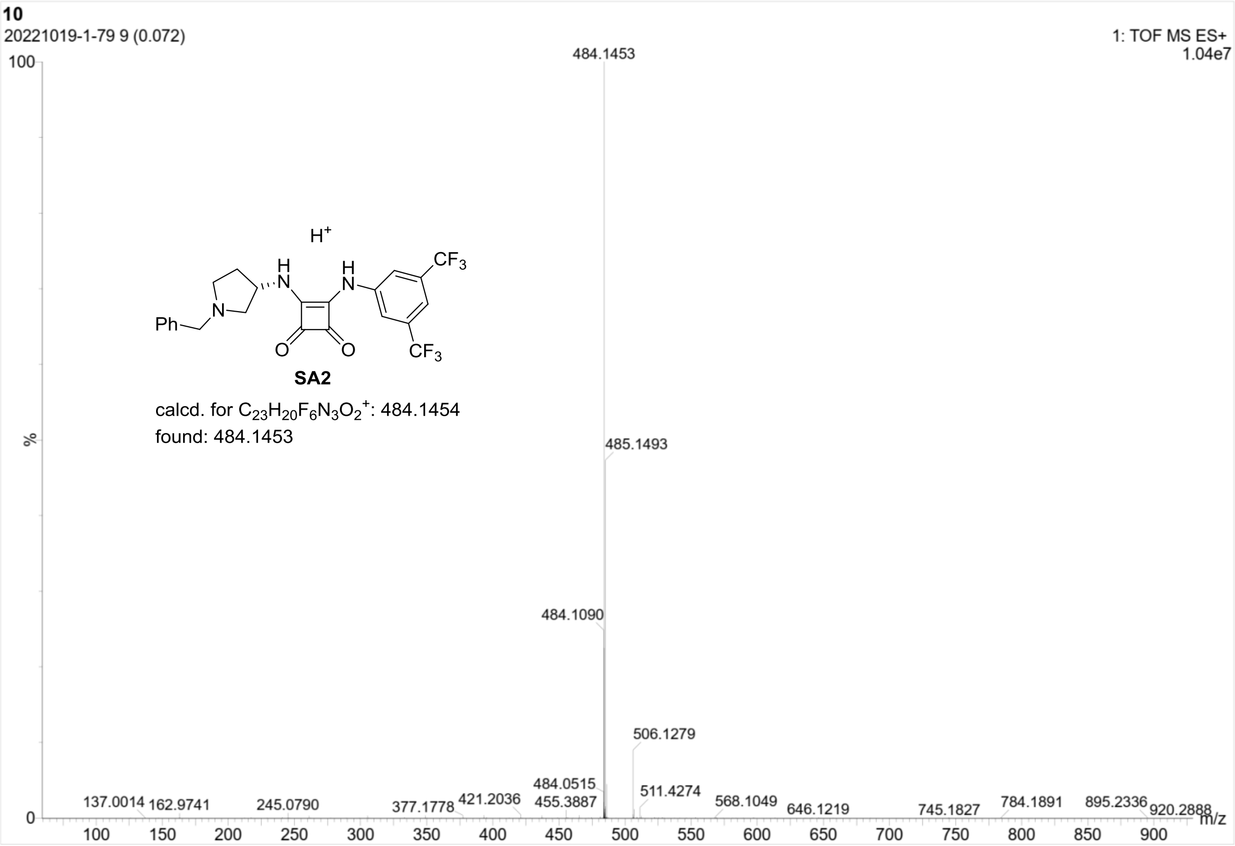
10
20221019-1-79 9 (0.072)
1: TOF MS ES+
1.04e7
484.1453
H+
CF3
CF3
Ph
SA2
calcd. for C23H20F6N3O2+: 484.1454
found: 484.1453
485.1493
484.1090
506.1279
484.0515
455.3887
511.4274
137.0014
162.9741
245.0790
377.1778
421.2036
568.1049
646.1219
745.1827
784.1891
895.2336
920.2888
%
m/z
100
0

HRMS of **SA2**

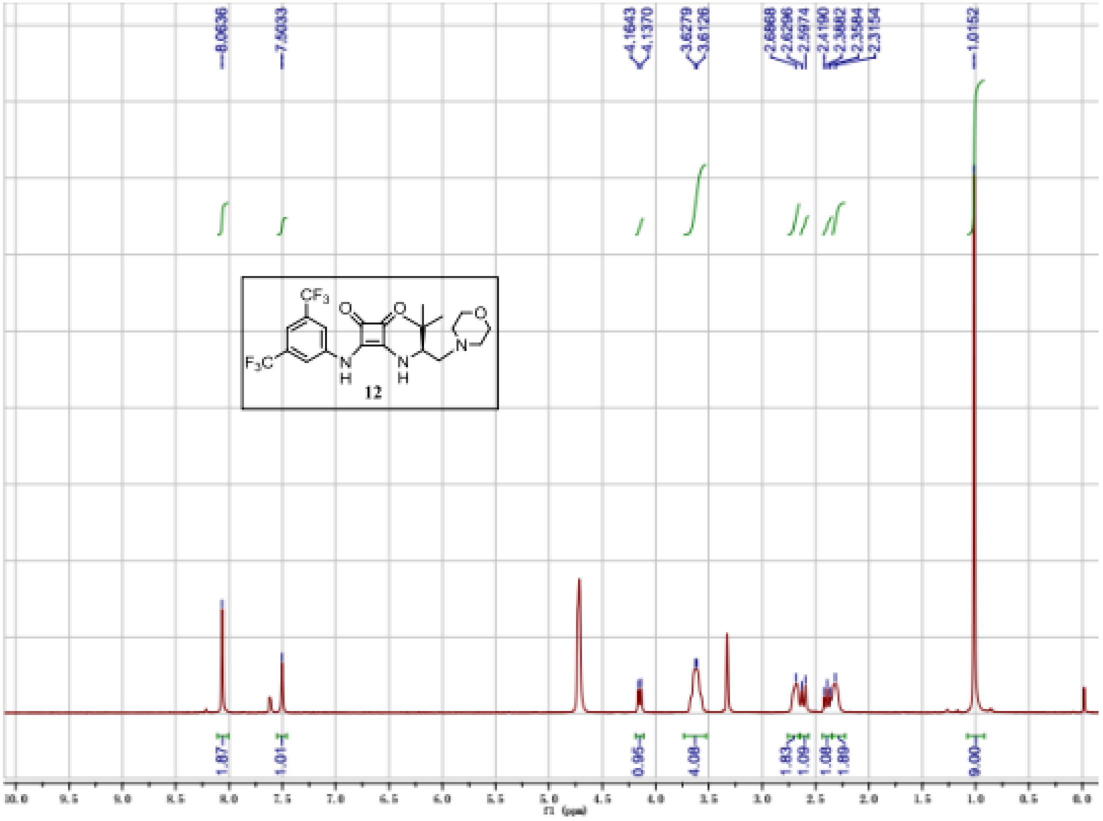


$^{1}$H NMR of **SA3**

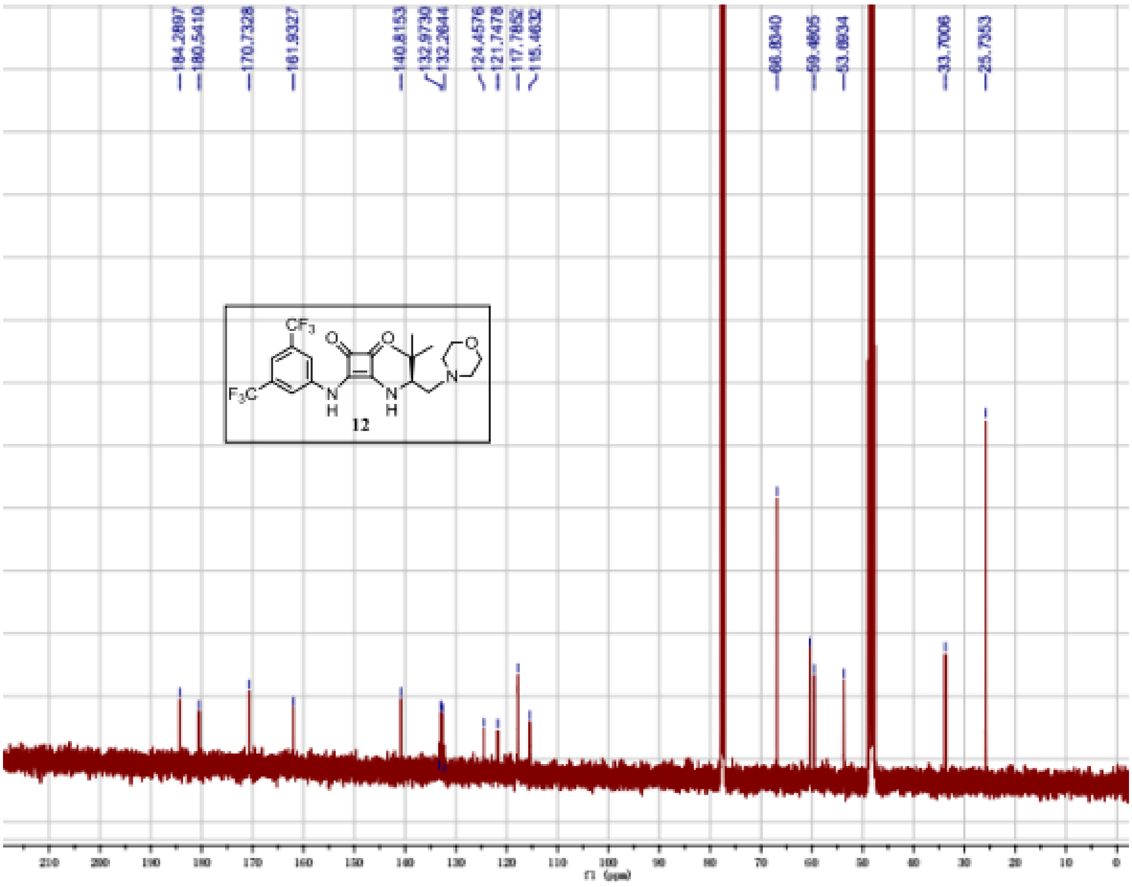


$^{13}$H NMR of **SA3**

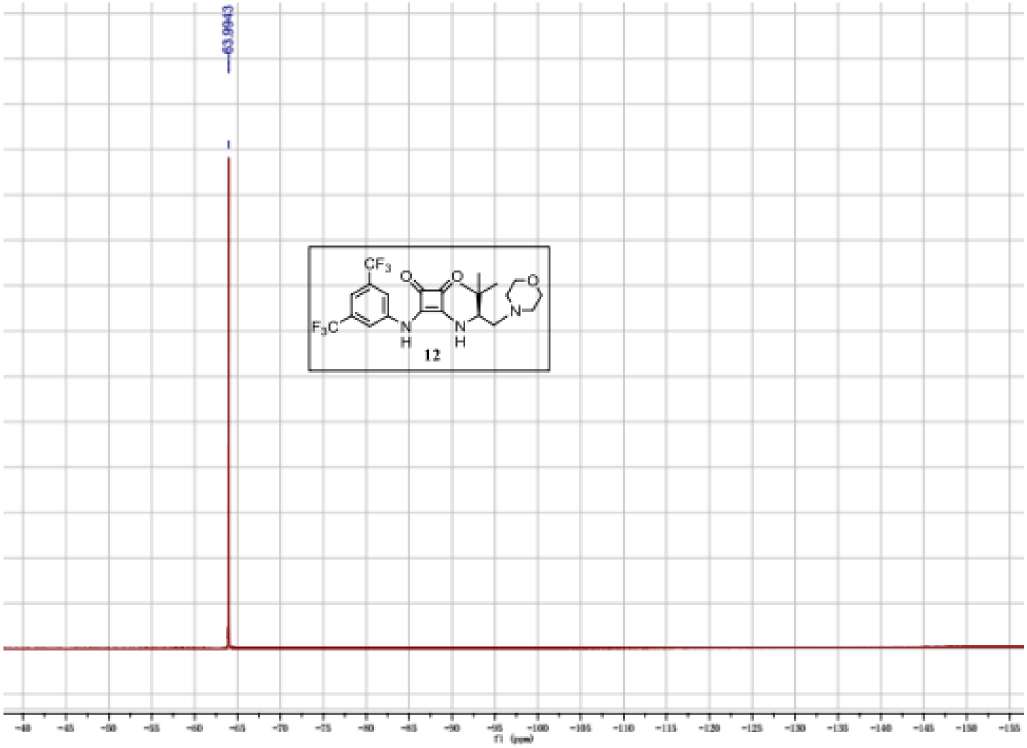


$^{19}$F NMR of **SA3**

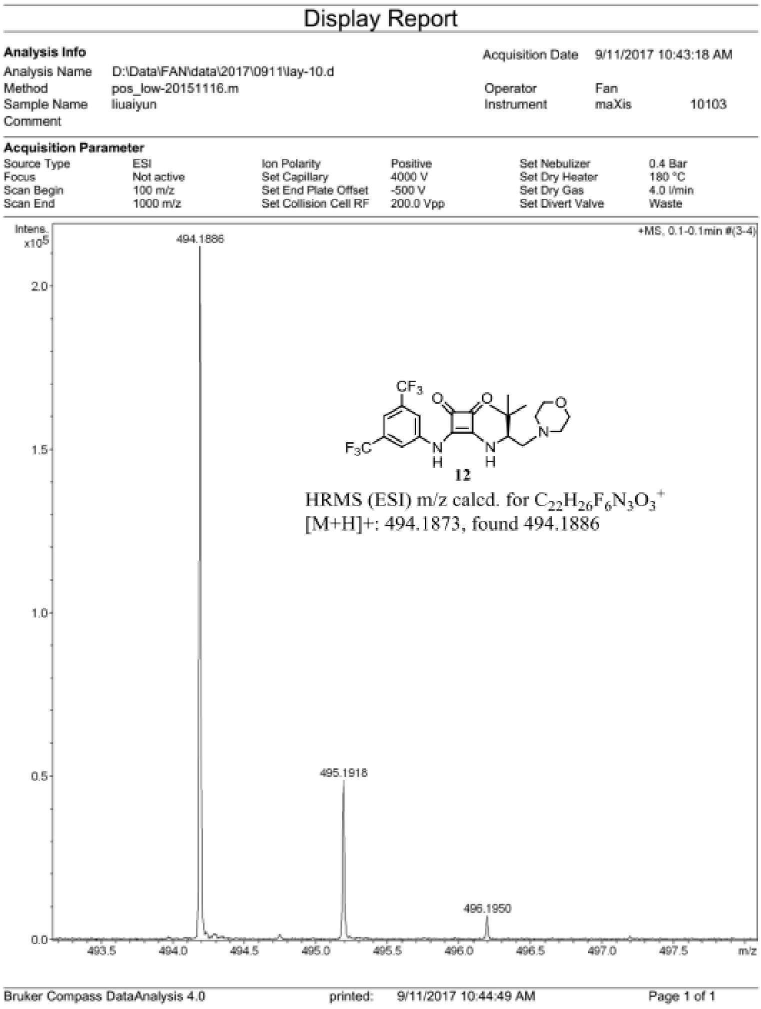
Display Report
Analysis Info
Analysis Name D:\Data\FAN\data\2017\0911\lay-10.d
Method pos_low-20151116.m
Sample Name liuaiyun
Comment
Acquisition Date 9/11/2017 10:43:18 AM
Operator Fan
Instrument maXis 10103
Acquisition Parameter
Source Type ESI
Focus Not active
Scan Begin 100 m/z
Scan End 1000 m/z
Ion Polarity Positive
Set Capillary 4000 V
Set End Plate Offset -500 V
Set Collision Cell RF 200.0 Vpp
Set Nebulizer 0.4 Bar
Set Dry Heater 180 °C
Set Dry Gas 4.0 l/min
Set Divert Valve Waste
Intens. x10^5
+MS, 0.1-0.1min #(3-4)
494.1886
495.1918
496.1950
m/z
12
HRMS (ESI) m/z calcd. for C22H26F6N3O3+ [M+H]+: 494.1873, found 494.1886
Bruker Compass DataAnalysis 4.0
printed: 9/11/2017 10:44:49 AM
Page 1 of 1

HRMS for **SA3**

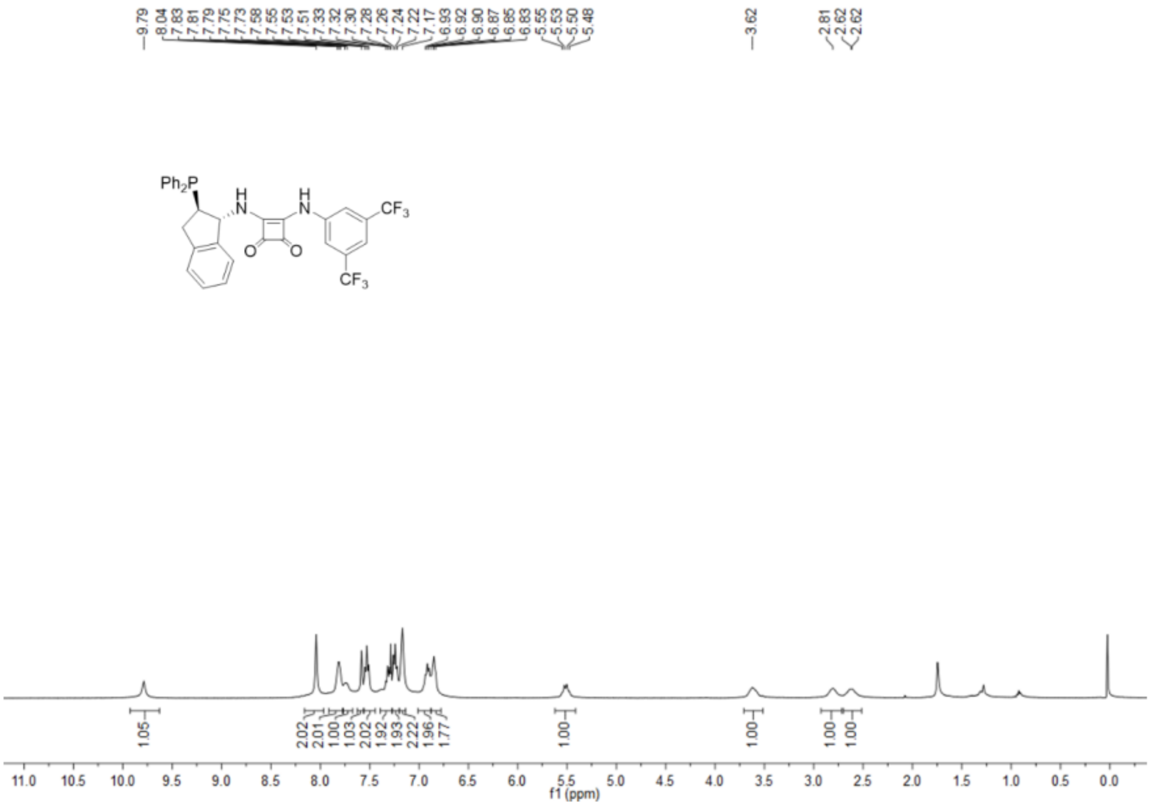

$^{1}$H NMR for **SA4**

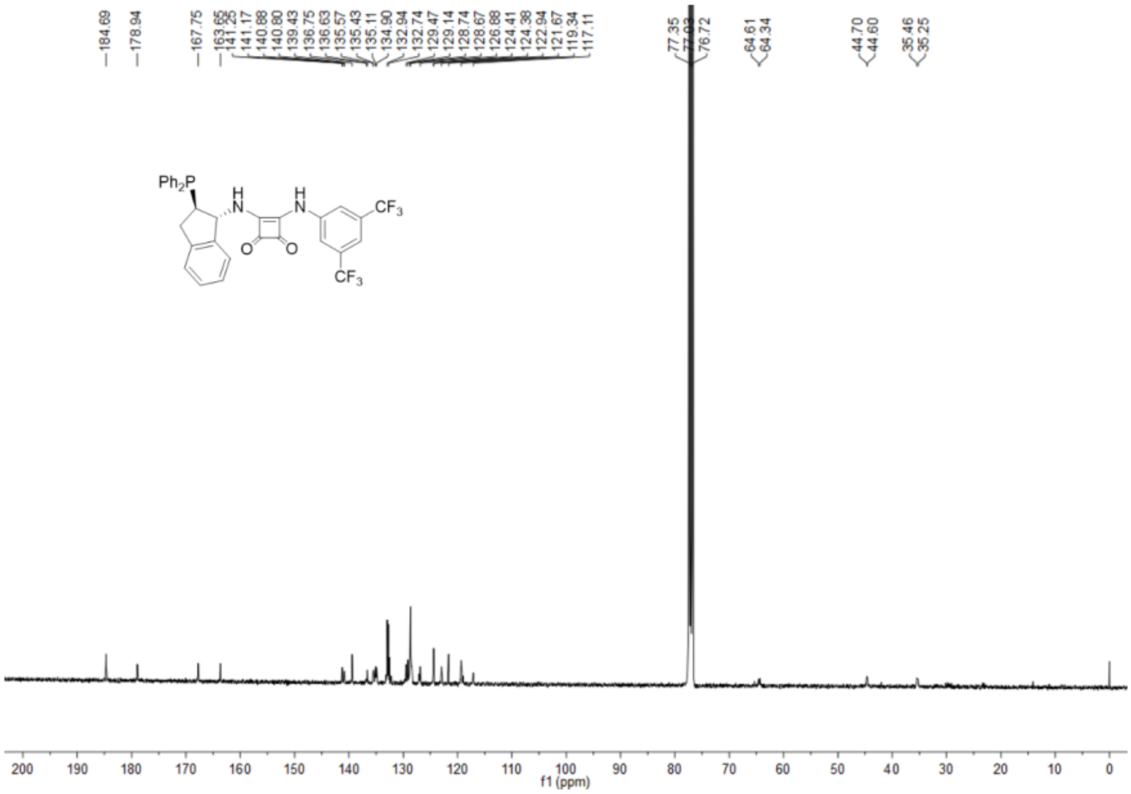

$^{13}$C NMR for **SA4**

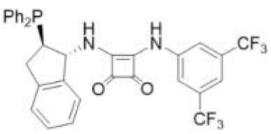


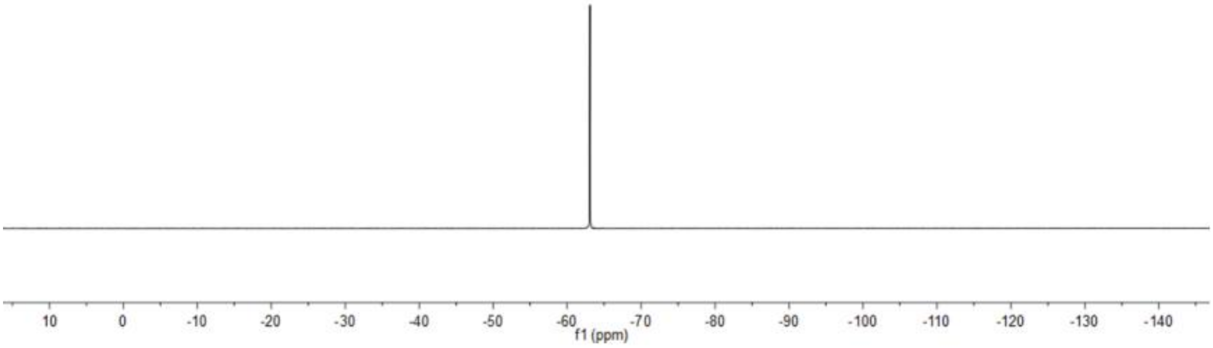


[19] F NMR for **SA4**

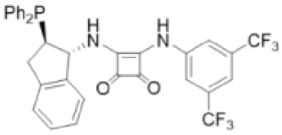


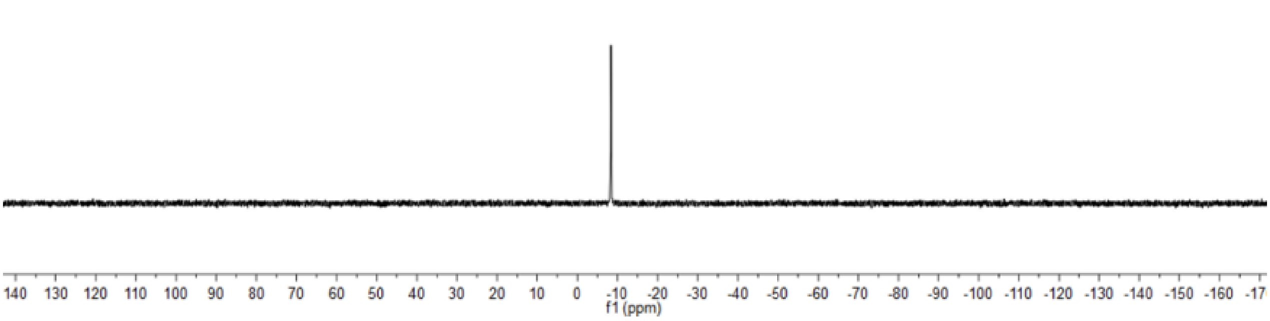


$^{31}$P NMR for **SA4**

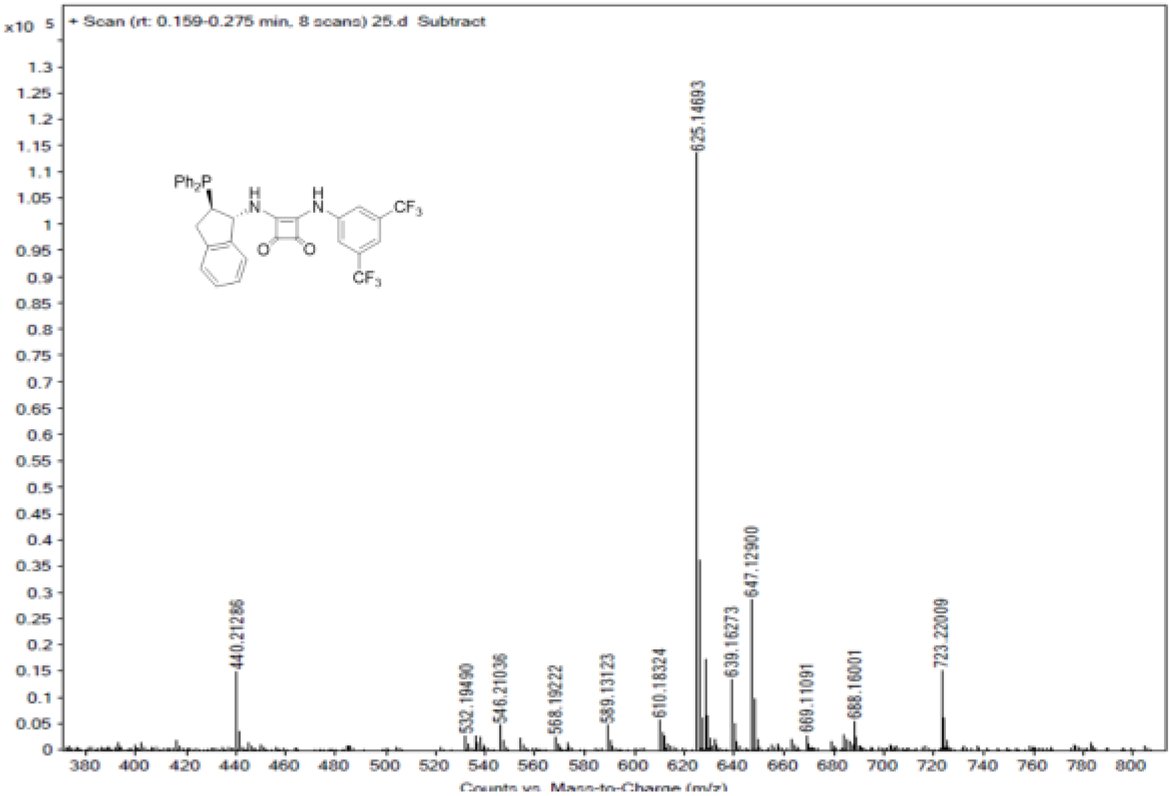


HRMS for **SA4**

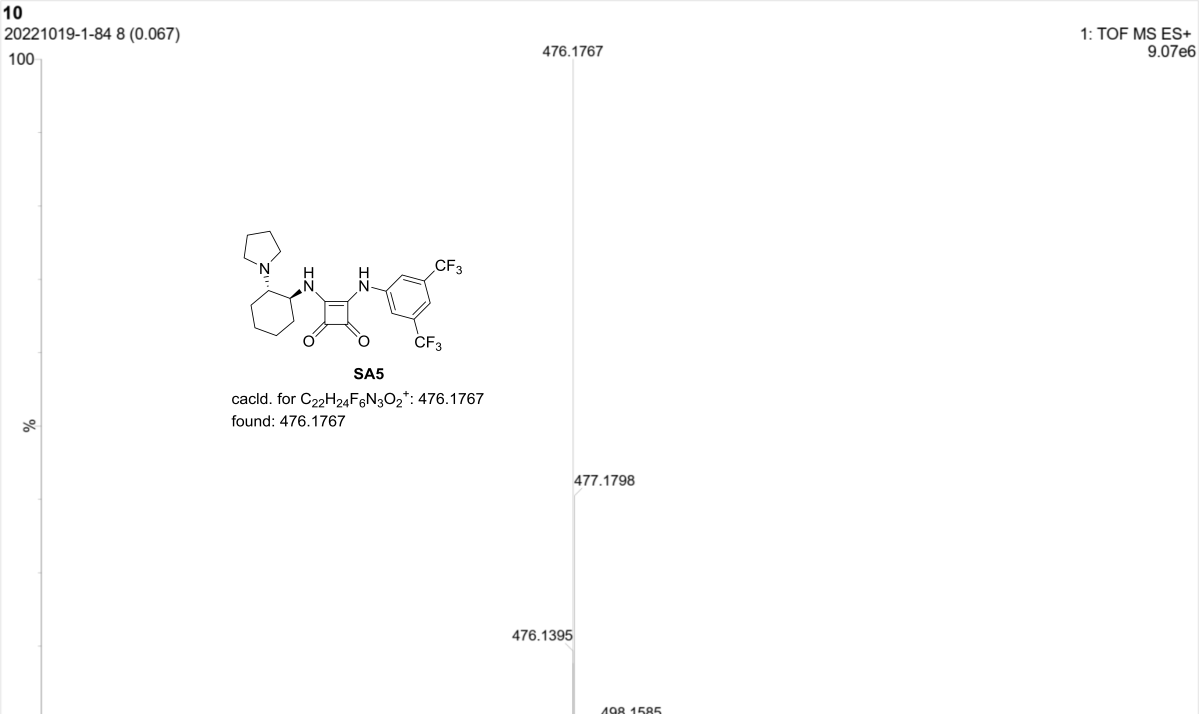


HRMS for **SA5**

**References**

[1] Konishi H.; Lam T.Y.; Malerich J.P.; Rawal V.H. Enantioselective $\alpha$-amination of 1,3-dicarbonyl compounds using squaramide derivatives as hydrogen bonding catalysts. *Org Lett* **2010**,12,2028-2031.

[2] Kowalczyk R.; Nowak A.E.; Skarzewski J. Organocatalytic asymmetric addition of aliphatic thiols to nitro olefins and nitrodienes. *Tetrahedron: Asymmetry* **2013**,24,505-514.

[3] Zhu J.; Cui D.; Li Y.; He J.; Chen W.; Wang P. Enantioselective amination of nitroolefins under base-free and water-rich conditions using chiral bifunctional phase-transfer catalysts. *Org Biomol Chem* **2018**,16,3012-3017.

[4] Li Y.; Cui D.; Zhu J.; Huang P.; Tian Z.; Jia Y.; Wang P. Bifunctional phase-transfer catalysts for fixation of $CO_2$ with epoxides under ambient pressure. *Green Chem*.**2019**, 21, 5231-5237.

**Supplementary Table S1** Anti-bacterial activity of SA compounds

| Compound/drug | MIC (µg/mL) | |
|---|---|---|
| | *S. aureus* (ATCC 29213) | MRSA (USA300) |
| Compound SA1 | 32 | 64 |
| Compound SA2 | 2-4 | 4 |
| Compound SA3 | 64 | 64 |
| Compound SA4 | >256 | >256 |
| Compound SA5 | 64 | 64 |
| Oxacillin | 2 | >256 |
| Ceftazidime | 4 | >256 |

**Supplementary Table S2** Primers of RT-PCR

| Gene | | Primer sequence (5'-3') |
|---|---|---|
| *norB* | Forward | GCTACACCATCAACAGATACAGC |
| | Reverse | ACGCCAAATGCTCCACCTAA |
| *ald* | Forward | TGTAGCTTGCACACCCGAAA |
| | Reverse | CAGCTTCCCATGCTTGTTCG |
| *tdcB* | Forward | AGGCTATGGGGCAAAGGTTA |
| | Reverse | CGCCAACTGGTACGATGACT |
| *gyrB* | Forward | ACAGGAATCGGTGGCGACTTTG |
| | Reverse | GCTCCATCCACATCGGCATCAG |
